# Genetic Diversity and Signatures of Selection for Thermal Stress in Cattle and Other Two *Bos* Species Adapted to Divergent Climatic Conditions

**DOI:** 10.3389/fgene.2021.604823

**Published:** 2021-02-03

**Authors:** Pedro H. F. Freitas, Yachun Wang, Ping Yan, Hinayah R. Oliveira, Flavio S. Schenkel, Yi Zhang, Qing Xu, Luiz F. Brito

**Affiliations:** ^1^Department of Animal Sciences, Purdue University, West Lafayette, IN, United States; ^2^Key Laboratory of Animal Genetics, Breeding and Reproduction, MARA – National Engineering Laboratory for Animal Breeding – College of Animal Science and Technology, China Agricultural University, Beijing, China; ^3^Lanzhou Institute of Husbandry and Pharmaceutical Sciences, Chinese Academy of Agricultural Sciences, Lanzhou, China; ^4^Centre for Genetic Improvement of Livestock, Department of Animal Biosciences, University of Guelph, Guelph, ON, Canada; ^5^College of Life Sciences and Bioengineering, School of Science, Beijing Jiaotong University, Beijing, China

**Keywords:** cold stress, climate resilience, genetic resources, heat stress, heat tolerance, selective sweep

## Abstract

Understanding the biological mechanisms of climatic adaptation is of paramount importance for the optimization of breeding programs and conservation of genetic resources. The aim of this study was to investigate genetic diversity and unravel genomic regions potentially under selection for heat and/or cold tolerance in thirty-two worldwide cattle breeds, with a focus on Chinese local cattle breeds adapted to divergent climatic conditions, Datong yak (*Bos grunniens*; YAK), and Bali (*Bos javanicus*) based on dense SNP data. In general, moderate genetic diversity levels were observed in most cattle populations. The proportion of polymorphic SNP ranged from 0.197 (YAK) to 0.992 (Mongolian cattle). Observed and expected heterozygosity ranged from 0.023 (YAK) to 0.366 (Sanhe cattle; SH), and from 0.021 (YAK) to 0.358 (SH), respectively. The overall average inbreeding (±SD) was: 0.118 ± 0.028, 0.228 ± 0.059, 0.194 ± 0.041, and 0.021 ± 0.004 based on the observed versus expected number of homozygous genotypes, excess of homozygosity, correlation between uniting gametes, and runs of homozygosity (ROH), respectively. Signatures of selection based on multiple scenarios and methods (*F*_ST_, HapFLK, and ROH) revealed important genomic regions and candidate genes. The candidate genes identified are related to various biological processes and pathways such as heat-shock proteins, oxygen transport, anatomical traits, mitochondrial DNA maintenance, metabolic activity, feed intake, carcass conformation, fertility, and reproduction. This highlights the large number of biological processes involved in thermal tolerance and thus, the polygenic nature of climatic resilience. A comprehensive description of genetic diversity measures in Chinese cattle and YAK was carried out and compared to 24 worldwide cattle breeds to avoid potential biases. Numerous genomic regions under positive selection were detected using three signature of selection methods and candidate genes potentially under positive selection were identified. Enriched function analyses pinpointed important biological pathways, molecular function and cellular components, which contribute to a better understanding of the biological mechanisms underlying thermal tolerance in cattle. Based on the large number of genomic regions identified, thermal tolerance has a complex polygenic inheritance nature, which was expected considering the various mechanisms involved in thermal stress response.

## Introduction

Climate change is a major concern around the world as it negatively impacts the welfare and sustainability of livestock production and, consequently, food security in a rapidly growing population (Polsky and Keyserlingk, 2017; Thamo et al., 2017; Henry et al., 2018; Bernabucci, 2019). Understanding the adaptation of different cattle (*Bos taurus*) breeds to local environments is of paramount importance for the design and optimization of breeding programs and conservation of genetic resources (Hoffmann, 2010; Mwai et al., 2015; Naskar et al., 2015). Domestication, followed by spatial dispersion, has resulted in the formation of multiple cattle breeds that are raised in a large range of environmental conditions and production systems (Ajmone-Marsan et al., 2010; Yurchenko et al., 2018). Consequently, long-term (natural and artificial) selection pressure has operated in the genomic regions that control traits for adaptive fitness and animal resilience (Kristensen et al., 2015; Berry, 2017).

Commercial cattle breeds are mainly used in intensive farming systems, with a limited number of traditional cattle breeds raised throughout the world (Kukučková et al., 2017; Mastrangelo et al., 2018; Upadhyay et al., 2019). In contrast, traditional cattle breeds have a long history of adaptation to their local environments (Mwai et al., 2015; Zhang et al., 2018). Whereas commercial cattle far exceed the traditional (or native) cattle breeds in terms of milk or meat production, the latter breeds have great cultural value, are usually adapted to local environmental and climatic conditions, and are sometimes genetically superior for specific production or functional traits (e.g., fertility, disease resistance, workability) compared to commercial cattle breeds (Medugorac et al., 2009; Mastrangelo et al., 2018; Upadhyay et al., 2019).

Cattle were domesticated in Asia and there is a wide variety of breeds adapted to different environmental conditions, which can be clustered into groups based on their geographical origin (Lei et al., 2006). For instance, Chinese cattle breeds are mainly composed by *Bos taurus taurus* (especially in Northern China) and *Bos taurus indicus* (mainly in the South of China) or hybrids between both subspecies (Li et al., 2013; Xu et al., 2019b). In addition to the *Bos taurus* species, domestic yak (*Bos grunniens*) and Bali cattle (BLI; *Bos javanicus*) have a substantial economic and social role in their respective habitat. In the mountainous regions of Asia, Datong yak (YAK) provides milk, meat, fiber and hide, and transportation (Wiener et al., 2003; Wiener, 2013). YAKs have the ability to survive in extremely harsh environments at high altitudes, overcoming hypoxia, severely cold winters, and cool moist summers, with limited feeding sources (Wiener, 2013; Chen et al., 2018). However, little is known about the specific adaptive mechanisms of YAKs. BLI cattle are found in Indonesia and Malaysia, and carries unique characteristics such as ability to survive dry seasons and higher heat tolerance, high conception rate, and greater resistance to diseases when compared to buffalo and cattle breeds (Noor et al., 2001; Syed-Shabthar et al., 2013). Other breeds around the world have also adapted to survive in extreme conditions, such as African cattle breeds that have adapted to high temperatures and food shortages (Mwai et al., 2015), and others that have adapted to extreme cold temperatures (Weldenegodguad et al., 2019).

Understanding how selection and evolution act on livestock genomes can benefit the optimization of breeding programs in order to improve economically important traits (Hiemstra et al., 2010; Mastrangelo et al., 2018), and especially animal resilience. For instance, a genomic evaluation for improved heat tolerance has been recently developed for Australian dairy cattle (Nguyen et al., 2016). There is an increased interest in identifying and exploring signatures of selection (also known as selective sweeps) and the genomic diversity resulting from adaptation to environment and artificial selective pressure (Igoshin et al., 2019; Osei-Amponsah et al., 2019; Upadhyay et al., 2019). Different approaches can be used to scan the genome for regions of homozygosity, as well as to estimate differences in the frequency of alleles or haplotypes between divergent populations or even generations within a population (Boschiero et al., 2018; Ceballos et al., 2018).

Several statistical methods have been proposed to identify signatures of selection, such as the fixation index (*F*_ST_) statistics (Wright, 1922), runs of homozygosity (ROH; Ku et al., 2010), and hapFLK (Fariello et al., 2013). ROH are genomic regions containing contiguous homozygous genotypes (Ceballos et al., 2018; Dixit et al., 2020). The *F*_ST_ is based on the measure of population differentiation due to locus-specific allele frequencies between populations (Wright, 1922; Maiorano et al., 2018) and is an alternative to analyze genetic differences across individuals (populations, breeds, or groups of animals) that may have been caused by opposing selection pressure (Beaumont, 2005; Willing et al., 2012). High *F*_ST_ values indicate local positive adaptation while low *F*_ST_ values suggest negative or neutral selection (Kullo and Ding, 2007). The hapFLK approach is based on haplotype differentiation between populations and it incorporates hierarchical structure of populations extended to account for haplotype structure in the samples (Fariello et al., 2013; Yurchenko et al., 2018). These methods have been applied in various livestock species [e.g., cattle (Randhawa et al., 2016; Cheruiyot et al., 2018), sheep (Moradi et al., 2012; Mcrae et al., 2014), goats (Brito et al., 2017; Onzima et al., 2018), pigs (Ai et al., 2013, 2014), chickens (Stainton et al., 2014; Walugembe et al., 2019)] and revealed multiple genomic regions associated with environmental adaptation (as reviewed by Randhawa et al., 2016). Therefore, the main objectives of this study were to investigate genetic diversity levels based on multiple metrics in cattle, with a focus on Chinese local breeds, and unravel genomic regions and candidate genes potentially under selection for thermal tolerance in cattle breeds adapted to divergent climatic conditions worldwide, domestic yak (Datong breed; *Bos grunniens*), and Bali (*Bos javanicus*) populations. To our best knowledge, no signatures of selection have been reported in the literature for some important Chinese cattle breeds [Yunnan Humped (DH), Dengchuan (DC), Wannan (WN), Wenling Humped (WL), Mongolian (MG), Sanhe (SH), Hazake (KZK), Xinjiang Brown (XIN)], which are unique genetic resources adapted to divergent climatic conditions. Furthermore, adding outgroups (closely related species) to the analyses can contribute to a better understanding of evolutive processes related to climatic adaptation that shaped cattle speciation, which has not been well investigated due to previous lack of genomic datasets.

## Materials and Methods

No Animal Care Committee approval was necessary for the purposes of this study, as all information required was obtained from pre-existing databases.

### Datasets

For the different analysis incorporated in this study different datasets were considered (specification of the populations used for the calculation of each diversity metric are described latter on). In summary, a total of 986 animals from 32 different cattle (*Bos taurus*) breeds, Datong yak (*Bos grunniens*) and Bali (*Bos javanicus*) were included in this study (Supplementary Table 1). Three sources of genotypes were used: (1) a set of 17 populations [DH, DC, WL, WN, Arabic Zebu (ZAR), BLI, Bangladesh zebu (BAD), Boran (BAN), Canchin (CAN), Creole from Guadalupe (CGU), East African Shorthorn zebu (EAZ), Gir (GIR), N’Dama (NDA), Senepol (SEN), Zebu Bororo (ZBO), Zebu from Madagascar (ZMA), and Zebu Fulani (ZFU)], considered as heat tolerant; (2) a set of 13 populations [SH, MG, KZK, XIN, Angus (ANG), Hereford (HFD), Holsteins (HOL), Jersey (JER), Brown Swiss (BSW), Kalmykian (KAL), Negra Andaluza (NAN), Santa Gertrudis (SGT), and Shorthorn (SHO)] considered as cold tolerant; and, (3) YAK, Busha (BUS), Yanbian (YAN), and Yakutian (YAKU) that are considered as resilient to cold and harsh environmental conditions. All animals were genotyped with the Illumina BovineSNP50K BeadChip (50K; Illumina, San Diego, CA, United States), except XIN and YAK animals that were genotyped using the GeneSeek GGP-HDV3 SNP panel (139K; GeneSeek, Lincoln, NE, United States). The 50K and 139K single nucleotide polymorphisms (SNP) panels contained 53,714 and 139,376 SNP, respectively. From the populations used in this study, the genomic information of 25 (see Supplementary Table 1) were downloaded from the Web-Interfaced next generation Database for genetic Diversity Exploration (Sempéré et al., 2015). A limitation of the other two *Bos* genus (YAK and BLI) datasets are the genomic datasets, in which genotyping platforms developed for cattle (a closely related *Bovidae* species) were used. This might have resulted in ascertainment bias (Lachance and Tishkoff, 2013; Malomane et al., 2018; Geibel et al., 2019) in the results of YAK and BLI, as discussed later. Genotypic quality control (QC) was implemented for all breeds together. SNP with minor allele frequency (MAF) lower than 0.01, animal and SNP call rate lower than 90%, and SNP located on the sex chromosomes or with unknown position in the genome were excluded from the analyses. Genotypic QC was performed using the software PLINK 1.9 (Purcell et al., 2007). The number of common overlapping SNP between SNP panels that remained after QC was 35,505. Several signatures of selection studies have successfully used similar SNP panel densities (e.g., Flori et al., 2012; Rothammer et al., 2013; Randhawa et al., 2014, 2016; Makina et al., 2015).

### Genetic Diversity Metrics

#### Heterozygosity

Observed heterozygosity (*H*_O_) per animal and within population was calculated based on the number of heterozygotes divided by the total number of genotypes. *H*_O_ values were compared to the expected heterozygosity under Hardy Weinberg Equilibrium (*H*_E_). Both metrics were calculated using the *– hardy* flag (Purcell et al., 2007; Chang et al., 2015).

#### Proportion of Polymorphic SNP (*P*_SNP_)

The *P*_SNP_ was calculated as the proportion of SNP with MAF greater than 1% within each breed, indicating the proportion of SNPs segregating in each population. Both *P*_SNP_ and MAF calculations were performed before the genotyping QC.

#### Average Pairwise Genetic Distance (D)

The average proportion of alleles shared between two individuals was calculated as $D_{\text{ST}}=\frac{\text{IBS}2+0.5*\mathrm{IBS1}}{m}$, where IBS1 and IBS2 are the number of loci that share 1 or 2 identical-by-state (IBS) alleles, respectively, and *m* is the total number of loci. The genetic distance between all pair-wise combinations of individuals was calculated as: *D* = 1 − *D*_ST_.

#### Runs of Homozygosity

Runs of homozygosity were identified using the *– homozyg* flag (Purcell et al., 2007; Chang et al., 2015). The following criteria were considered to define a ROH segment: (i) scanning window of 50 SNP across the genome; (ii) minimum density of 50 kb/SNP; (iii) minimum ROH length of 500 kb (Xu et al., 2019a), and, (iv) maximum gap between consecutive homozygous SNP of 100 kb. To investigate genomic regions under possible selection, non-overlapping ROH segments between the heat and cold adaptation groups, and that were shared at least between 50% of the animals in the same breed were selected and compared with the other signature of selection metrics.

#### Inbreeding Coefficients

Four different measurements of genomic inbreeding were calculated for all cattle populations and YAK: (1) based on the observed versus expected number of homozygous genotypes (*F*_EH_), (2) genomic inbreeding based on excess of homozygosity (*F*_HOM_), (3) correlation between uniting gametes (*F*_U_), and (4) ROH-based inbreeding (*F*_ROH_). *F*_EH_, *F*_HOM_, and *F*_U_ were calculated using all genotyped animals and SNP that remained after the QC performed individually for each population. *F*_ROH_ was calculated as the genome length covered by ROH divided by the total genome length covered by SNPs across all 29 autosomes. Different inbreeding metrics are expected to differ as they capture complementary populational diversity characteristics (Meuwissen et al., 2020).

#### Linkage Disequilibrium (LD)

The extent of LD (measured as *r*^2^) was calculated for each breed group using the *–r2* flag (Purcell et al., 2007), as following (Sved, 1971):

$$\text{LD}=\frac{D^{2}}{\text{f}\left(\text{A}\right)\text{f}\left(\text{a}\right)\text{f}\left(\text{B}\right)\text{f}\left(\text{b}\right)}$$

where f(AB), f(A), f(a), f(B), and f(b) are observed frequencies of AB, A, a, B, and b, respectively, and *D*^2^ = [f(AB) − f(A)^∗^f(B)]^2^. Within-population quality control was used to calculate LD (or *r*^2^) for each breed. The LD decay was then analyzed for the following different genetic distance classes between SNP pairs (0, <10, <20, <40, <60, <100, <200, <500, <1,000 kb).

#### Consistency of Gametic Phase

Consistency of gametic phase was determined by calculating the square root of the LD values (*r*^2^) and assigning the appropriate sign based on the calculated disequilibrium (*D*) metric, as used in the calculation of LD. The D values were calculated using the *–dprime-signed* option (Purcell et al., 2007; Chang et al., 2015). Thereafter, the consistency of gametic phase was assumed as the Pearson correlation coefficient between each two breed-group pair, using the signed-squared-root values. The breakdown in the consistency of gametic phase across distances was determined based on the same bins described above. Only SNP in common (after within-population QC) among the cattle populations were used to calculate consistency of gametic phase.

#### Ancestral and Recent Effective Population Size (*N*e)

The LD combined with distance between markers can be used to estimate the approximate *N*e at a given time point in the past. To estimate *N*e through time, the formula as described by Sved (1971) was used:

$$Ne={\left(\left(\frac{1}{E\left(r^{2}\right)}\right)-1\right)}^{*}\left(\frac{1}{4c}\right)$$

where, *N*e is the effective population size, and *r*^2^ is the average *r*^2^ value at a given distance *c*. Each genetic distance (c) corresponds to a value of t generations in the past. This value was calculated as $t=\frac{1}{2c}$ (Hayes et al., 2003). The *N*e in generation 5 was assumed as the recent *N*e and ancient *N*e was investigated up to 500 generations in the past.

For all the above-mentioned metrics 35,505 SNPs from 312 individuals from eight Chinese cattle breeds (DH, DC, WL, WN, SH, MG, KZK, and XIN) and YAK population were used. These metrics were only calculated to these populations as all the other datasets were extracted from public databases and similar studies have been published elsewhere (e.g., Chen et al., 2016; Boushaba et al., 2018; Edea et al., 2018).

#### Principal Component Analysis (PCA)

For the PCA and genomic population tree (described below) all individuals were considered (*n* = 986) from the 34 populations (Supplementary Table 1). In order to better visualize the genetic similarity among populations, PCA was assessed using the *–pca* flag (Purcell et al., 2007; Chang et al., 2015). Two scenarios were analyzed: (1) including all the 34 populations; and (2) considering only the 32 cattle populations (i.e., excluding YAK and BLI).

#### Genomic Population Tree

The neighbor-joining algorithm was used to plot population trees using pair-wise Reynolds distance between populations (Fariello et al., 2013). The genomic population tree was created using all genome-wide SNP that passed the QC. The software PLINK 1.9 (Purcell et al., 2007; Chang et al., 2015) was used for calculating observed and expected heterozygosity, proportion of polymorphic SNP, genetic distance, ROH, genomic inbreeding coefficients, LD, gametic phase, and PCA.

### Signatures of Selection Analyses

#### Fixation Index

To identify population-specific loci under positive selection, *F*_ST_ was calculated for each of the 35,505 informative SNP along the genome using different contrasting groups to estimate the differences in allelic frequencies. In brief, *F*_ST_ was calculated as the squared deviation of the average frequency in a specific population from the average frequency across all populations divided by the allele frequency variance (*p*^∗^*q*, in which *p* and *q* are the frequencies of each alternate SNP alleles). In order to identify putative genomic regions under selection, the analyses were performed by grouping the cattle breeds in divergent clusters defined as heat, mild cold, and extreme cold tolerant populations. Preliminary analyses with balanced number of individuals per group were performed, but similar results were observed (results not shown). Therefore, the results with the complete datasets are presented here. Genomic regions under a mean plus four SD threshold were considered as candidate regions. To identify putative genome regions under selection, the analyses were performed under four scenarios of contrasting models:

(1) **Groups based on climate adaptation (*F*_**ST**_1):** The groups (*n* = 3) included all populations and were created based on climate adaptation: heat adaptation (DH, DC, WL, WN, ZAR, BLI, BAD, BAN, CAN, CGU, EAZ, GIR, NDA, SEN, ZBO, ZMA, and ZFU), mild cold/thermoneutral adaptation (SH, MG, KZK, XIN, ANG, HFD, HOL, JER, BSW, KAL, NAN, SGT and SHO) and harsh cold adaptation (YAK, BUS, YAN, and YAKU).
(2) **Groups based on climate adaptation and breeding purpose excluding YAK and BLI populations (*F*_**ST**_2):** The groups (*n* = 3) included all populations mentioned for *F*_ST_1, but YAK and BLI were excluded.
(3) **Most climatic-divergent individual breeds (*F*_**ST**_3):** The three most climatic-divergent populations (one for each group described above) based on *F*_ST_ values were compared against each other. The populations were DH (heat group), HOL (mild cold/thermoneutral group), and YAKU (harsh cold group).

#### HapFLK

The hapFLK method was used for calculation of distance matrices and the estimation of hapFLK as described by Fariello et al. (2013). Three scenarios were considered and included all populations (Supplementary Table 1). For the first scenario, YAK was considered as an outgroup; for the second BLI was considered as an outgroup; and for the third scenario there was no outgroup and only cattle breeds (32 breeds; Supplementary Table 1) were included. A Reynolds distance matrix was calculated to estimate the hierarchical population structure within each population set. The fastPHASE (Scheet and Stephens, 2006) cross-validation procedure was used to determine the number of underlying latent states. A total of 21 haplotype clusters were needed to capture the total haplotype diversity.

#### Candidate Genes and Enriched Function Analyses

The positional candidate genes located in genomic regions containing relevant SNP based on *F*_ST_, ROH, and HapFLK analyses were mapped using the Biomart tool (Kinsella et al., 2011), embedded in the Ensembl Genes database version 99^1^. Based on the start and end chromosomal positions, important genomic regions were further investigated to identify biological processes related to climatic adaptation and to define prospective functional genes. Complete gene functions were obtained from the National Center for Biotechnology Information Database^2^ based on the latest *Bos taurus* reference genome ARS-UCD1.2 (Rosen et al., 2020) and from the Animal QTL database (Hu et al., 2018). Biological functions and Kyoto Encyclopedia of Genes and Genomes (KEGG) pathways (Kanehisa, 2000; Kanehisa et al., 2016, 2018) in which the identified candidate genes are involved were assessed using the Database for Annotation, Visualization and Integrated Discover tool (DAVID v6.8; Huang et al., 2008a, b). Four different scenarios were analyzed during the enriched function analyses considering high classification stringency taking into consideration: (1) all candidate genes found in all methods together (heat and cold adaptation; SCEN1), (2) candidate genes identified in methods based on heat adaptation (SCEN2), (3) candidate genes identified in methods based on cold adaptation (SCEN3), and (4) candidate genes identified for YAK (SCEN4).

## Results

### Genetic Diversity Metrics

The following genetic diversity metrics were estimated from eight Chinese cattle breeds (DH, DC, WL, WN, SH, MG, KZK, and XIN) and YAK population. As previously mentioned, these metrics were only calculated to these populations as all the other datasets were extracted from public databases and similar studies have been published elsewhere (e.g., Chen et al., 2016; Boushaba et al., 2018; Edea et al., 2018).

#### Heterozygosity

*H*_O_ ranged from 0.023 (YAK) to 0.366 (SH). The average *H*_O_ was lower than the *H*_E_ for DH, DC, Wannam, and MG populations, indicating slightly lower genetic diversity levels than expected.

#### Proportion of Polymorphic SNP

**FIGURE 1:**
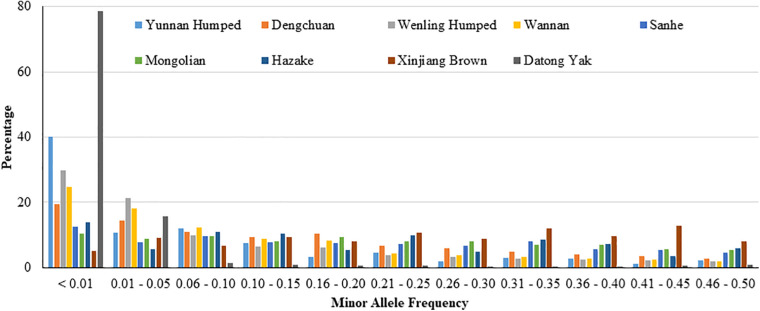
Distribution of single nucleotide polymorphisms (SNP percentage) by minor allele frequency ranges in eight Chinese cattle breeds and Datong yak.

The *P*_SNP_ was high in all analyzed populations (ranged from 0.659 to 0.992), except in YAK (0.197). The distribution of SNP percentage by MAF range is shown in Figure 1. In general, the distribution of SNP percentage was variable across the different populations, especially for YAK, which showed the highest value for MAF less than 0.01 and the lowest values for the other MAF ranges.

#### Average Pairwise Genetic Distance

D ranged from 0.016 (YAK) to 0.290 (MG) (Table 1). Higher values indicate greater within-population genetic variation (heterogeneity).

**TABLE 1:** Genetic diversity metrics estimated within each one of the eight Chinese cattle breeds and Datong yak.

	Yunnan Humped	Dengchuan	Wenling Humped	Wannan	Sanhe	Mongolian	Hazake	Xinjiang Brown	Datong Yak
**Species**	Bos taurus sspp.*	Bos taurus sspp.*	Bos taurus sspp.*	Bos taurus sspp.*	*Bos taurus taurus*	*Bos taurus taurus*	*Bos taurus taurus*	*Bos taurus taurus*	*Bos grunniens*
**Purpose**	Work	Work	Work	Work	Various	Various	Various	Meat/Dairy	Various
**Adaptation**	Heat	Heat	Heat	Heat	Cold	Cold	Cold	Cold	Cold
**Coat color**	Yellow	Yellow	Yellow	Yellow	Red and white	Mixed	Mixed	Brown	Black
**Coat color pattern**	Solid with light and dark pattern	Solid with light and dark pattern	Solid with light and dark pattern	Solid with light and dark pattern	Spotted	Mixed	Mixed	Solid with light and dark pattern	Solid
**Coat hair length**	Short	Short	Short	Short	Long in winter	Long in winter	Long in winter	Long in winter	Long
**Sample size**	16	31	31	31	32	35	16	35	45
***P*_SNP_**	0.659	0.894	0.783	0.839	0.988	0.992	0.964	0.949	0.197
***H*_O_**	0.166	0.256	0.186	0.210	0.366	0.351	0.357	0.329	0.023
***H*_E_**	0.170	0.258	0.185	0.211	0.358	0.357	0.346	0.318	0.021
***D***	0.149	0.216	0.155	0.179	0.287	0.290	0.285	0.256	0.016
**LD**	0.189 (0.112)	0.136 (0.089)	0.198 (0.085)	0.159 (0.081)	0.183 (0.123)	0.145 (0.114)	0.168 (0.109)	0.206 (0.091)	0.545 (0.109)
***F*_HOM_ ± SD**	−0.113 (0.045)	−0.097 (0.031)	−0.158 (0.022)	−0.155 (0.031)	0.453 (0.018)	0.326 (0.021)	0.357 (0.021)	0.421 (0.067)	0.024 (0.003)
***F*_EH_ ± SD**	0.504 (0.064)	0.233 (0.045)	0.442 (0.102)	0.371 (0.067)	−0.095 (0.040)	−0.049 (0.054)	−0.066 (0.009)	0.017 (0.068)	0.935 (0.008)
***F*_U_ ± SD**	0.220 (0.050)	0.0942 (0.035)	0.174 (0.053)	0.139 (0.038)	0.181 (0.053)	0.137 (0.057)	0.100 (0.011)	0.228 (0.070)	0.480 (0.004)
***F*_ROH_ ± SD**	0.028 (0.003)	0.011 (0.002)	0.029 (0.004)	0.012 (0.001)	0.002 (0.001)	0.022 (0.005)	0.001 (0.001)	0.034 (0.009)	0.087 (0.011)

Adap., adaptation; Samp., sample size; P_SNP_, proportion of polymorphic SNP; H_O_ and H_E_, expected and observed heterozygosity, respectively; D, average pairwise genetic distance; LD, average linkage disequilibrium between adjacent SNP; SD, standard deviation; F_HOM_, F_EH_, F_U_, and F_ROH_, inbreeding coefficients based on excess of homozygosity, on the observed versus expected number of homozygous genotypes, on the correlation between uniting gametes, and on the runs of homozygosity, respectively. *Mixed composition (Bos taurus taurus × Bos taurus indicus).

#### Inbreeding Coefficients

Four different approaches to estimate genomic inbreeding coefficients were used. The average genomic inbreeding coefficients estimated using different approaches are shown in Table 1. The overall average genomic inbreeding (±SD) coefficients were: 0.254 ± 0.028 (*F*_HOM_), 0.250 ± 0.051 (*F*_EH_), 0.194 ± 0.041(*F*_U_), and 0.021 ± 0.004 (*F*_ROH_). The average inbreeding coefficients differed among cattle breeds and YAK had the highest values for *F*_HOM_, *F*_U_, and *F*_ROH_ metrics. Table 2 presents the Pearson correlations among the different measures of genomic inbreeding coefficients. In general moderate to high correlations were observed among all methods (0.57–0.89), with exception of *F*_EH_ and *F*_HOM_, which surprisingly had a negative correlation (−0.71).

**TABLE 2:** Pearson correlation among inbreeding coefficients in eight Chinese cattle breeds and Datong yak.

	*F*_HOM_	*F*_U_	*F*_ROH_
***F*_EH_**	−0.78	0.69	0.89
***F*_HOM_**		−0.07	0.73
***F*_U_**			0.12

*F_HOM_, F_EH_, F_U_, and F_ROH_ inbreeding coefficients based on excess of homozygosity, on the observed versus expected number of homozygous genotypes, on the correlation between uniting gametes and on the runs of homozygosity, respectively.*

#### Runs of Homozygosity

**FIGURE 2:**
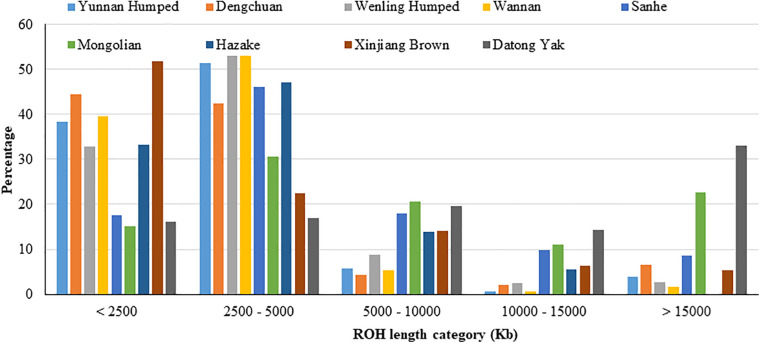
Proportion of runs of homozygosity segments in each length category for eight Chinese cattle breeds and Datong yak.

The proportion of ROH segments in each length category for the nine populations are shown in Figure 2 and Supplementary Table 1. The majority of ROH segments was of short length (i.e., segments were shorter than 2,500 kb or between 2,500 and 5,000 kb), indicating ancient inbreeding. DH, DC, WN, and XIN had the highest proportion of short segments compared to the other populations. Only a small proportion of ROH segments were longer than 15,000 kb, except for YAK, which had the highest proportion of longer ROH segments, and MG cattle population. The KZK population had the highest proportion of short ROH segments and, in contrast, no ROH segment higher than 15,000 kb.

A descriptive summary of ROH is presented in Table 3. The average number of ROH segments for each animal within breed ranged from 2.57 ± 1.4 (KZK) to 163.5 ± 16.39 (YAK), with a maximum of 247 segments in a YAK. The average length of genome contained within ROH segments ranged from 3,505 kb ± 2,409.51 (KZK) to 14,669 kb ± 16,059.5 (YAK). The animal with the longest proportion of its genome characterized as ROH was also observed in the YAK population (123,041 kb). The average number of SNP in ROH run per population ranged from 81.28 ± 100.79 (WN) to 664.9 ± 705.51 (YAK), with minimum and maximum values of 50 and 5,398 SNP, respectively. The average SNP density (SNP per kb) was similar for all populations (∼43 SNP/kb), except for XIN and YAK which were ∼21 SNP/kb. For all populations, the proportion of homozygous sites was higher than 97%.

**TABLE 3:** Descriptive analysis of the runs of homozygosity per population in eight Chinese cattle breeds and Datong yak.

		Population								
		DH	DC	WL	WN	SH	MG	KZK	XIN	YAK
**N_SEG_**	**Mean**	42	14.13	60	36.55	8	4.69	2.57	53.91	163.5
	**SD**	8.47	5.37	11.69	10.21	5.23	5.06	1.4	19.75	16.39
	**Min**	30	6	30	14	0	0	0	19	137
	**Max**	65	31	75	52	20	24	5	89	247
**KB**	**Mean**	4,425	4,513	3,944	3,505	7,018	10,673	3,901	4,497	14,669
	**SD**	8,081.06	6,148.62	3,717.87	4,678.48	8,540.82	11,950.8	2,409.51	5,240.94	16,059.5
	**Min**	1,683	1,680	1,608	1,494	1,719	1,610	1,798	500	502
	**Max**	107,178	40,170	39,035	91,311	53,723	76,286	12,436	44,445	123,041
**N_SNP_**	**Mean**	100.76	103.01	90.57	81.28	159.8	238.8	91.81	218.2	664.9
	**SD**	170.93	131.75	81.53	100.79	182.58	256.094	53.51	245.93	705.51
	**Min**	50	51	50	50	50	51	52	50	50
	**Max**	2254	857	857	1894	1149	1639	298	2022	5398
**Density**	**Mean**	43.14	42.47	42.95	42.56	42.47	43.25	42.01	21.15	21.62
	**SD**	4.16	4.43	4.14	4.29	4.03	4.3	4.68	4.89	3.58
	**Min**	30.75	30.85	30.34	28.73	30.84	31.34	32.1	20.91	3.55
	**Max**	49.99	49.97	49.98	49.96	49.87	49.99	49.57	42.21	49.742
**P_HOM_**	**Mean**	0.97	0.98	0.97	0.97	0.98	0.99	0.98	0.99	0.99
	**SD**	0.01	0.01	0.01	0.01	0.02	0.01	0.01	0.01	0.01

*NSEG, average number of segments for the individual declared homozygous; KB, average of total number of kb contained within homozygous segments; NSNP, average number of SNP in run; PHOM, proportion of sites homozygous. DH, Yunnan Humped; DC, Dengchuan; WL, Wenling Humped; WN, Wannan; SH, Sanhe; MG, Mongolian; KZK, Hazake; XIN, Xinjiang Brown; YAK, Datong yak.*

#### Linkage Disequilibrium

**FIGURE 3:**
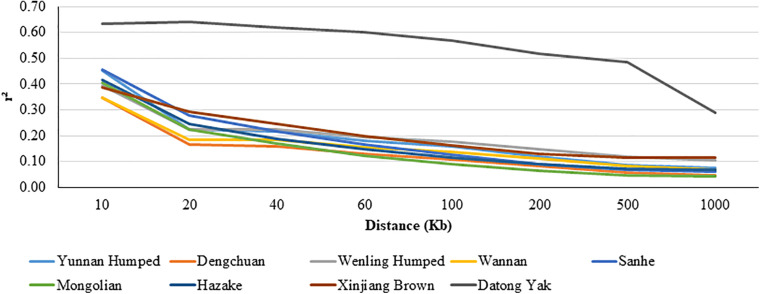
Average linkage disequilibrium (LD) at given distances for eight Chinese cattle breeds and Datong yak.

The LD decay pattern for all populations is shown in Figure 3. In general, the highest LD was observed for YAK [ranged from 0.642 (at 0.02 Mb) to 0.290 (at 1 Mb)] and DH [ranged from 0.453 (at 0.01 Mb) to 0.074 (at 1 Mb)]. The LD decay pattern for WN was similar to DC cattle. WN [ranged from 0.346 (at 0.01 Mb) to 0.069 (at 1 Mb)] and DC [ranged from 0.347 (at 0.01 Mb) to 0.045 (at 1 Mb)] had the lowest LD values.

#### Consistency of Gametic Phase

**FIGURE 4:**
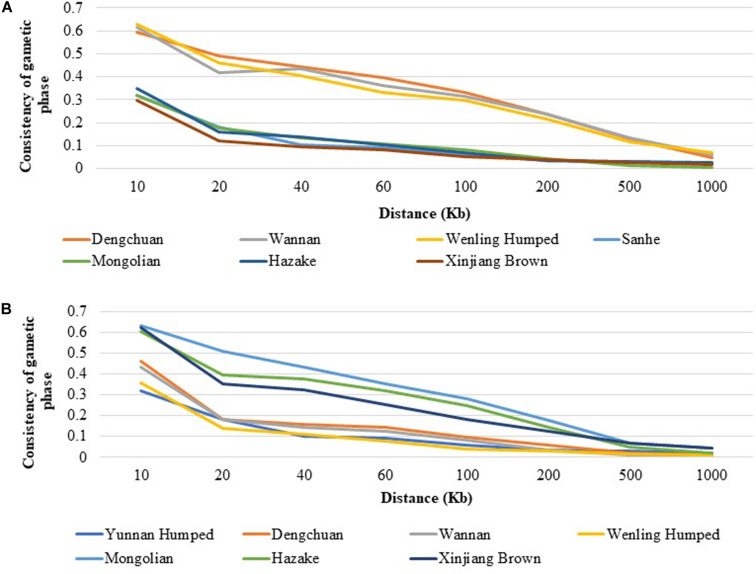
Consistency of gametic phase at given distances between **(A)** Yunnan Humped and seven Chinese cattle breeds, and **(B)** Sanhe and seven Chinese cattle breeds.

As an example, the consistency of gametic phase between DH and SH with the other Chinese cattle breeds are shown in Figure 4. Among all populations DC, WN, and Weiling Humped had the highest consistency of gametic phase with DH (Figure 4A) at all analyzed distances, ranging from 0.594 (at 10 Kb) to 0.047 (at 1,000 Kb), from 0.618 (at 10 Kb) to 0.061 (at 1,000 Kb), and from 0.628 (at 10 Kb) to 0.067 (at 1,000 Kb), respectively. On the other hand, MG and XIN populations had the lowest consistency of gametic phase with DH, ranging from 0.319 (at 10 Kb) to 0.004 (at 1,000 Kb) and from 0.298 (at 10 Kb) to 0.018 (at 1,000 Kb), respectively. Similar trends to DH have been observed for DC and WN with the other populations. It was observed that the consistency of gametic phase values between SH and the other cattle breeds (Figure 4B) have similar values after 100 Kb distance. The taurine breeds have similar consistency of gametic phase values with each other and the same was observed with the hybrid populations. The consistency of gametic phase between all population pairs are shown in Supplementary Table 2.

#### Ancestral Effective Population Size

The historical *N*e ranged from 3,032 (MG breed) at 500 generations ago to 47 (KZK) at five generations ago. The *N*e five generations ago (considered as recent *N*e) were quite variable across populations, ranging from 147 (DC) to 47 (KZK). YAK had the smallest *N*e 500 generations ago (*N*e = 195) and 66 five generation ago. The *N*e values from 5 to 500 generations ago are shown in Table 4.

**TABLE 4:** Ancestor effective population size based on linkage disequilibrium (*r*^2^), calculated from generation 5 to 500 for eight Chinese cattle breeds and Datong yak.

Generations ago	Population								
	Yunnan Humped	Dengchuan	Wenling Humped	Wannan	Sanhe	Mongolian	Hazake	Xinjiang Brown	Datong yak
**500**	1,367	2,113	1,188	1,609	1,750	3,032	1,952	1,328	194
**250**	909	1,414	710	1,009	1,237	2,488	1,263	844	117
**100**	527	830	375	557	668	1,637	629	383	53
**75**	461	769	314	494	558	1,445	498	268	60
**50**	317	533	222	347	376	971	341	203	109
**25**	179	258	205	180	196	497	166	129	171
**10**	112	179	118	84	86	189	56	87	118
**5**	76	147	111	78	69	128	47	77	66

#### Principal Component Analysis

**FIGURE 5:**
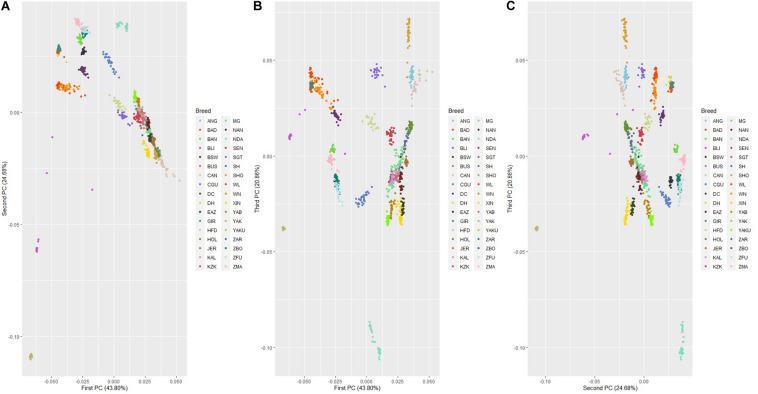
Principal component decomposition of the genomic relationship matrix colored by population*. Letters in the figure represent the decomposition of the: **(A)** first and second; **(B)** first and third; and **(C)** second and third principal components, respectively (*see Supplementary Table 1 for abbreviations).

The genetic relationship among 32 cattle breeds, YAK and BLI populations revealed by PCA are shown in Figure 5. The first, second, and third principal components explained 43.80%, 24.68%, and 20.86% of the total variability among cattle breeds, YAK and BLI, respectively. In general, all three components show a clear differentiation of groups adapted to heat and cold climatic conditions. Populations adapted to heat were grouped together and the same occurred with those adapted to cold, and YAK and BLI were clustered as two separate group from the other populations, as expected. Breeds with similar development were clustered together such as XIN and BSW. Two clear clusters were observed for the Zebu breeds (*Bos indicus*; ZBO, ZFU, ZMA, and EAZ) and another group of Chinese cattle breeds adapted to heat (DH, DC, WL, and WN), which have small contributions from *Bos taurus indicus* from crossing over centuries ago. The PCA plots considering only the 32 cattle populations are shown in Supplementary Figure 1. The first, second, and third principal components explained 58.61%, 24.82%, and 19.94% of the total variability among cattle breeds, respectively. It was observed that the populations remained grouped into common clusters based on adaptation to heat or cold stress, despite of their sub-species. As in the first PCA scenario, there were clusters for grouping XIN and BSW, Zebu breeds and Chinese heat adapted breeds. In the two PCA scenarios, specially between first and second, and second and third PC, it is also possible to see that breeds adapted to the cold/thermoneutral temperatures are located together (e.g., HOL, JER, and NAN).

#### Genomic Population Tree

**FIGURE 6:**
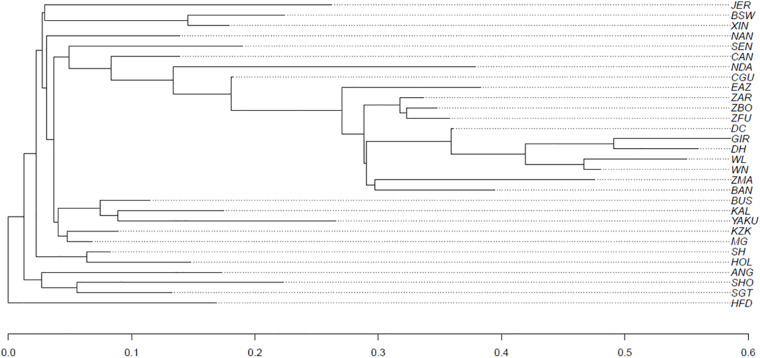
Genomic population tree comparing the genomic distance between 32 cattle breeds*. (*see Supplementary Table 1 for abbreviations).

The genomic population tree (constructed based on whole-genome SNPs) for 32 cattle breeds is presented in Figure 6. In summary, the populations were grouped according to their specific sub-species, i.e., hybrid (*Bos taurus indicus* × *Bos taurus taurus*), taurine (*Bos taurus taurus*) or indicine (*Bos taurus indicus*) breeds were clustered separately. Also, populations with similar development background were clustered together (e.g., XIN and BSW).

### Signatures of Selection Analyses

#### Fixation Index

**FIGURE 7:**
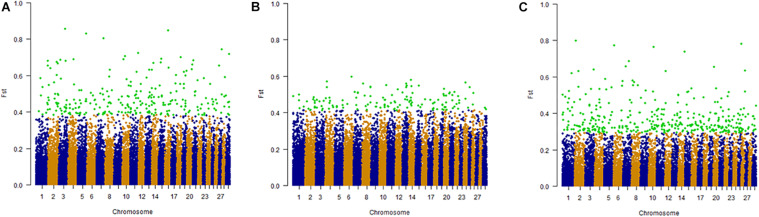
SCENARIO 1: Whole genome scans for signatures of selection using the *F*_ST_ approach for 32 cattle breeds and yak, considering climate adaptation. Letters in the figure represent: **(A)** Heat adapted populations, **(B)** Mild cold/thermoneutral adapted populations, and **(C)** Harsh cold adapted populations.

High *F*_ST_ values indicate potential positive selection while low *F*_ST_ values suggest negative or neutral selection. Considering 32 cattle breeds, YAK and BLI populations there were several genomic regions potentially under selection in at least one of the populations studied. These selective sweeps were distributed on all 29 autosomal chromosomes. In scenario 1 (*F*_ST_1), where we contrasted climate adaptation groups (*n* = 3), we observed 303 significant SNP and 3 shared SNP were found between the groups (one between mild cold/thermoneutral and heat, and two between heat and harsh cold). The highest number of SNP identified was on BTA1 (15 SNP) in the harsh cold adapted group and the lowest was on BTA1 (1 SNP) in the cold adapted group. In *F*_ST_2, which contrasted climate adaptation groups (*n* = 3), but excluding YAK and BLI from the comparison, 858 significant SNP were identified. Thirty-one SNP were shared between harsh cold and the heat adapted group, and a total of 827 unique SNP were observed. The highest number of SNP identified was on BTA1 (40 SNP) in the harsh cold adapted group and one SNP were found to be significant in BTA 3 in the cold/thermoneutral group. For *F*_ST_3, where the most climatic-divergent breeds were compared, 987 significant SNP were observed. For the mild cold/thermoneutral and heat groups there were seven overlapping SNP, 37 between heat and harsh cold groups and 11 between mild cold/thermoneutral and harsh cold groups. The highest number of SNP identified was on BTA2 (62 SNP) in DH and the lowest was on BTA28 (7 SNP) in the MG population. As an example of the *F*_ST_ process, Figure 7 shows scans for signatures of selection based on *F*_ST_1, considering climate adaptation and including YAK and BLI genotypes. Supplementary Figures 2, 3 show the genome scans for *F*_ST_2 and *F*_ST_3, respectively.

#### HapFLK

**FIGURE 8:**
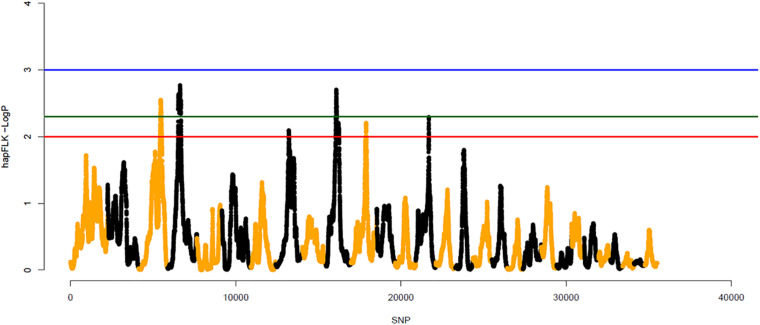
Whole genome scans for signatures of selection using the haplotype based hapFLK metric considering 32 cattle breeds and Yak as outgroup (first scenario). Odd and even numbered chromosomes are shown in yellow and black, respectively. SNP number is given on the *x*-axis, and the genome-wide threshold corresponding to *P* < 0.001, *P* < 0.005, and *P* < 0.01 is shown as horizontal blue, green and red lines, respectively.

The hapFLK method enables the identification of genomic regions of outlying differentiation between populations while accounting for their hierarchical structure (Fariello et al., 2013). Significant peaks were identified using the hapFLK metric for assessing haplotype differentiation between populations. Peaks with *p*-values < 0.005 were considered significant. These peaks were located in BTA3, BTA4, BTA10, and BTA14, for the first scenario, using YAK as an outgroup. In the second scenario, which had BLI population as outgroup, peaks were also found on BTA3, BTA4, BTA10, and BTA14, as well as on BTA15. In the third scenario, which considered only the 32 cattle populations and no outgroup, there were also significant peaks in BTA3, BTA4, BTA10, and BTA14. The highest peaks for the three scenarios were located on BTA10. The haplotype based hapFLK metric and −log (*p*-values) for the whole genome are shown in Figure 8 for the first scenario and Supplementary Figures 4, 5 for the second and third scenario, respectively.

#### Runs of Homozygosity

The highest number of ROH segments in the cattle populations were found for XIN and Weiling Humped (1,887 and 1,804, respectively) and the lowest (36) was found in KZK. The highest number of ROH segments were observed in BTA6 in XIN (179 ROH segments) and BTA1 in the Weiling Humped (153 ROH segments). The lowest number of ROH segments was observed in BTA28 in the DC, SH, and MG breeds (2 ROH segments). No ROH segments were found for KZK in BTA7, BTA15, BTA16, BTA18, BTA19, BTA26, BTA27, BTA28 and BTA29. The number of ROH segments observed in YAK was substantially greater (7,358 ROH segments) in comparison to the cattle populations. The number of ROH segments per chromosome in YAK ranged from 145 (BTA25) to 487 (BTA5).

### Positively Selected Regions

The genomic regions with *F*_ST_ values greater than the mean plus four SD for *F*_ST_1, *F*_ST_2, and *F*_ST_3; hapFLK *p*-values smaller than 0.005, and regions in ROH were further investigated to identify potential genes under positive selection for thermal tolerance. A summary of the number of regions identified and the number of genes for each method and scenario is presented in Table 5. The largest number of candidate genes were found based on ROH and *F*_ST_1, 8,702 and 1,206 candidate genes, respectively. On the other hand, the lowest number (30) was found for hapFLK when considering YAK as an outgroup. Some of the genes located in at least two different metrics were considered as relevant genes for further investigation. For instance, some of these genes are related to heat shock proteins (e.g., *DNAJB13*), oxygen transport (e.g., *HBB* and *HBG*; Neupane et al., 2017), mitochondrial DNA maintenance (e.g., *PIF1*; Foury and Kolodynski, 1983), metabolic activity (e.g., *HSPE1*; Kumar et al., 2015), feed intake (e.g., *SNORA70*; Pitt et al., 2018), carcass conformation (e.g., *SNORA70*; Pitt et al., 2018; *PLCL1*; Shin et al., 2015), fertility and reproduction traits (e.g., *RBPMS2*, *OAZ2*; Fernández et al., 2019; *IL6ST*; Song et al., 2009; *IGSF8*; Glazar and Evans, 2009), and olfactory receptors (e.g., *OR51A7*; Olivieri et al., 2016).

**TABLE 5:** Summary of the number of regions identified and the number of genes (inside brackets) under positive selection for individual (diagonal) method/scenario and between methods/scenarios (off diagonal).

	*F*_ST_1	*F*_ST_2	*F*_ST_3	hapFLK (outgroup – Yak)	hapFLK (outgroup – Bali)	hapFLK (no outgroup)	ROH
***F*_ST_1**	888 (1,201)	101 (171)	569 (872)	1 (2)	–	–	986 (1,012)
***F*_ST_2**		661 (757)	107 (173)	3 (3)	1 (3)	–	697 (715)
***F*_ST_3**			911 (1,215)	2 (3)	2 (3)	2 (4)	1,203 (1,194)
**hapFLK (outgroup – Yak)**				24 (30)	19 (24)	–	178 (27)
**hapFLK (outgroup – Bali)**					37 (41)	–	
**hapFLK (no outgroup)**						17 (35)	192 (29)
**ROH**							4,202 (4,972)

*See “Materials and Methods” for definition of methods (F_ST_, hapFLK or ROH) and scenarios (F_ST_ 1 to 3, hapFLK outgroup or no outgroup).*

Several novel candidate genes were found when combining different methods. Two candidate genes were identified in YAK [*RBPMS2* (BTA10: 45.31–45.33 Mb) and *PIF1* (BTA10: 45.27–45.27 Mb)] and one was present only in MG cattle [*ATG16L2* (BTA15: 52.35–52.36 Mb)]. These same two candidate genes present in YAK were identified between *F*_ST_2 and hapFLK. Two novel candidate genes were also identified based on hapFLK and *F*_ST_3, including *OAZ2* (BTA10: 45.36–45.37 Mb) and *CSNK1G1* (BTA10: 45.66–45.81 Mb). Larger number of overlapping regions were identified when comparing scenarios based either on *F*_ST_ or hapFLK to the ROH-based approach. For instance, the gene *SNORA70* (BTA11: 99.006–99.007 Mb) was identified in DC cattle based on ROH and *F*_ST_1. All genomic regions and candidate genes are reported in Supplementary Tables 4, 5 for individual method and overlapping regions among methods, respectively, and in Supplementary Table 6 are the candidate genes identified between the three methods (or scenarios).

The functionally enriched categories of candidate genes are shown in Supplementary Tables 7–10. The main functionally enriched categories with the respective genes within each cluster are shown in Table 6. Considering the first scenario (SCEN1), the candidate genes associated with thermal stress were significantly enriched into 116 functional categories (*P* < 0.05) including hemoglobin complex (GOTERM_CC), oxygen binding (GOTERM_MF), oxygen transport (GOTERM_BP), epidermal growth factor-like domain (INTERPRO), carbohydrate biosynthetic process (GOTERM_BP), steroid hormone receptor and steroid hormone receptor activity (INTERPRO and GOTERM_MF), folate receptor and folate receptor-like (INTERPRO and GOTERM_MF), folic acid transporter activity and folic acid binding (INTERPRO and GOTERM_MF), biosynthesis of unsaturated fatty acids (KEGG_PATHWAY), sperm capacitation and fertilization (GOTERM_BP and UP_KEYWORDS), immunoglobulin-like domain and immunoglobulin subtype (INTERPRO), and immunoglobulin I-set and immunoglobulin subtype 2 (INTERPRO). In the second scenario (SCEN2), where candidate genes identified in the signatures of selection analyses in the heat adaptation groups were analyzed, 65 significantly enriched functional categories were identified including hemostasis and blood coagulation (UP_KEYWORDS), platelet activation (GOTERM_BP), fertilization (UP_KEYWORDS), sperm capacitation and single fertilization (GOTERM_BP), immunoglobulin-like domain and immunoglobulin subtype (INTERPRO), sugar/inositol transporter, general substrate transporter and sugar transporter (INTERPRO), myosin head [motor domain (INTERPRO)], myosin complex (GOTERM_CC), myosin (UP_KEYWORDS), steroid hormone receptor and nuclear hormone receptor [ligand-binding-core (INTERPRO)], steroid hormone receptor activity (GOTERM_MF), collagen triple helix repeat and collagen trimer (INTERPRO and GOTERM_CC), collagen (UP_KEYWORDS) and respiratory chain (UP_KEYWORDS).

**TABLE 6:** Main enriched function categories and candidate genes for thermal tolerance in eight local Chinese cattle and Datong yak populations.

Category*	Term	Genes	p-value
SCEN1–GOTERM	Hemoglobin complex and oxygen transport	*ALB, HBE2, HBM, HBZ, HBB, HBE4, HBE1, HBG, HBQ1*	< 0.00001
SCEN1–GOTERM	Carbohydrate biosynthetic process	*CHST11, CHST14, CHST8, CHST9*	0.008
SCEN1–GOTERM	Folic acid transport	*FOLR1, FOLR2, FOLR3*	0.010
SCEN1–INTERPRO	Immunoglobulin	*BCAM, CADM2, CD3G, TYRO3, CNTN2, FGFR4, FLT1, HMCN1, HMCN2, HSPG2, IGSF9, IL1RL1, MUSK, MYOM2, LRIG1, MYOM3, MYPN, NFASC, NTRK2, OPCML, PALLD, PTPRS, ROR2, ROBO2*	0.035
SCEN1–KEGG	Unsaturated fatty acid biosynthesis	*ACOX1, ACOT2, ACOT4, LOC512541*	0.046
SCEN1–GOTERM	Sperm capacitation	*ACRBP, BSP1, BSP3, BSP5*	0.033
SCEN1–GOTERM	Steroid hormone receptor activity	*RORA, RORB, HNF4A, NR1I2, NR4A3, PPARD, THRB, THRA*	0.039
SCEN2–INTERPRO	Immunoglobulin complex	*CD3E, CD3G, CD7, SPEG, AMIGO1, BCAM, CADM2, CNTN3, FGFR4, IGSF10, IL1RL1, IL1RL2, IL1R1, IL18R1, IL18RAP, KALRN, LRFN5, LRIG1, MFAP3, MYOM3, MYPN, NECTIN2, PTPRM, SEMA3A, SEMA3C, SEMA3D, SEMA4D, SEMA7A, TMEM25, UNC5B, ZPBP2*	0.017
SCEN2–GOTERM	Fertilization	*BSP1, BSP3, BSP5*	0.031
SCEN2–UP_KEYWORDS	Hemostasis and blood coagulation	*F3, FGA, FGG, VWF*	0.026
SCEN2–INTERPRO	General substrate and sugar transporter	*SLC2A1, SLC2A10, LOC107131287*	0.047
SCEN2–GOTERM	Platelet activation	*FGA, FGG, VWF*	0.04
SCEN2–GOTERM	Myosin complex	*MYO1D, MYO1E, MYO5A, MYO5C*	0.041
SCEN2–GOTERM	Steroid hormone receptor activity	*RORA, RORB, HNF4A, THRA*	0.040
SCEN2–GOTERM	Collagen	*C1QTNF7, COL4A3, COL9A2, COL8A2, COL11A1*	0.063
SCEN2–UP_KEYWORDS	Respiratory chain	*NDUFS3, NDUFB6, CYCS, CYCT*	0.065
SCEN3–GOTERM	Myosin complex	*MYO1E, MYO3A, MYO5A, MYO6*	0.014
SCEN3–GOTERM	Innate immunity	*CYLD, MX1, MX2, ALCAM, LBP, NOD2, SPON2, TICAM1*	0.040
SCEN3–GOTERM	Cell–cell adhesion	*LASP1, OLA1, RAB11B*	0.080
SCEN4–KEGG	Salivary and acid gastric secretion	*ADCY4, ADCY8, BST1, CALM1, ITPR2, PLCB1, PRKCG*	0.003
SCEN4–GOTERM	Hemoglobin complex and oxygen transport	*ALB, HBM, HBZ, HBQ1*	0.006
SCEN4–INTERPRO	Immunoglobulin complex	*TYRO3, BCAM, CADM2, IGSF9, LRIG1, LOC100335205, NFASC, NECTIN2, PALLD, ROR2, ROBO2, SLAMF9, SEMA3D, SEMA7A, TMEM25, UNC5D*	0.009
SCEN4–KEGG	Prolactin signaling pathway	*MAPK8, MAPK9, PIK3R3, PRLR*	0.029
SCEN4–GOTERM	Myosin complex	*MYO1D, MYO3A, MYO5A*	0.034
SCEN4–GOTERM	Steroid hormone receptor activity	*RORA, THRB, THRA*	0.045

*SCEN1: all candidate genes found in all methods together (heat and cold adaptation); SCEN2: candidate genes identified in methods based on heat adaptation; SCEN3: candidate genes identified in methods based on cold adaptation; SCEN4: candidate genes identified for Datong yak.

Considering the third scenario (SCEN3), where candidate genes identified in the signatures of selection analyses in the cold adaptation groups were analyzed, 36 significantly enriched functional categories were identified including myosin head [motor domain (INTERPRO)], myosin complex (GOTERM_CC), innate immunity and innate immune response (UP_KEYWORDS and GOTERM_BP), immunity (UP_KEYWORDS), cell–cell adhesion and cell–cell adherens junction (GOTERM_BP and GOTERM_CC), and immunoglobulin I-set and immunoglobulin V-set (INTERPRO). Lastly, we analyzed candidate genes identified in the signatures of selection analyses for YAK (SCEN4) and 35 significantly enriched functional categories were identified including salivary secretion and gastric acid secretion (KEGG_PATHWAY), oxygen binding (GOTERM_MF), hemoglobin complex (GOTERM_CC), oxygen transport (UP_KEYWORDS), oxygen transport activity (GOTERM_MF), immunoglobulin subtype 2 and immunoglobulin-like domain (INTERPRO), myosin head [motor domain (INTERPRO)], myosin complex (GOTERM_CC), secondary metabolites biosynthesis, transport, and catabolism (COG_ONTOLOGY), steroid hormone receptor and steroid hormone receptor activity (INTERPRO and GOTERM_MF), and prolactin signaling pathway (KEGG_PATHWAY).

## Discussion

### Genetic Diversity Metrics

#### Population Characterization and *P*_SNP_

In general, the distribution of SNP percentage was approximately constant by MAF bins (Figure 1), and the *P*_SNP_ was high in the majority of the eight Chinese cattle breeds (DH, DC, WL, WN, SH, MG, KZK, and XIN) and YAK population studied in this section (Table 1). These results indicate the appropriateness of the SNP panels used for genomic studies in these populations as well as substantial genetic diversity, which could be due to the fact that these local breeds have not undergone intensive artificial selection, as experienced in commercial and worldwide spread populations. The higher *P*_SNP_ value found for taurine compared to hybrid breeds is in agreement with the literature (Lin et al., 2010; Edea et al., 2013). For instance, Edea et al. (2013) studying Korean Hanwoo cattle (*Bos taurus taurus*) also found high *P*_SNP_ (0.952). YAK was an exception, both for the distribution of SNP percentage and for the proportion of polymorphic SNP. These differences between YAK and the cattle breeds may be due to the fact that YAK was not included in the development of the SNP chip panel and the fact that YAK and cattle are different species (*Bos gruniens and Bos taurus*), even though closely related in the evolutionary tree. To minimize and avoid this potential issue, a SNP chip panel could be developed specifically for YAKs. Alternatively, genotyping-by-sequencing (GBS) could be employed (Qiu et al., 2015), which can be performed at a low cost per sample and tend to avoid ascertainment bias. The candidate genes obtained based on the scenarios including YAKs are biologically reasonable based on its known adaptation processes. Therefore, the results obtained are useful and worth reporting in this study.

#### Heterozygosity and Genetic Distance

Heterozygosity measures the level of genetic variation within a population. In this context, the levels of *H*_O_ and *H*_E_ were moderate to high (>0.166; Table 1), except for YAK where the values were 0.023 and 0.021 for *H*_O_ and *H*_E_, respectively. Similar levels of *H*_O_ and *H*_E_ were also reported by Zhang et al. (2018), studying XIN (*H*_O_ = 0.313 and *H*_E_ = 0.288) and MG (*H*_O_ = 0.331 and *H*_E_ = 0.332) cattle breeds. Similar heterozygosity values (*H*_O_ = 0.340 and *H*_E_ = 0.341) compared to those found for taurine breeds in this study have been reported in Australian HOL cattle (Zenger et al., 2007) as well as in African cattle breeds (*H*_O_ = 0.28–0.33 and *H*_E_ = 0.29–0.34; Boushaba et al., 2018). *H*_O_ was lower than *H*_E_ in the DH, DC, WN, and MG breeds indicating increased inbreeding and reduced genetic diversity levels (Sharma et al., 2016). The differences in heterozygosity levels observed among populations, especially for YAK, can be partially explained by the SNP array design, which did not include all the cattle populations evaluated in this study and therefore, ascertainment bias might have influenced the estimates. It is worth noting that cattle SNP chip panels have been used for genotyping animals from other *Bovidae* species (e.g., bison, water buffalo, YAK), which generated very relevant results (e.g., Michelizzi et al., 2011; Wu et al., 2012; Oleński et al., 2015).

The *D* value ranged from 0.149 to 0.290 in the eight Chinese cattle populations (Table 1), suggesting variation on the levels of genetic diversity within each population. For YAK, this value was lower, indicating lower genetic distance between individuals or it may be due to the fact of the SNP chip panel used. The results for genetic distance corroborate with the PCA results, where low genetic variation between individuals was found within each cattle breed and practically no variation was found between YAK individuals. The PCA showed that some of the populations were more clearly separated while others were clustered more closely. The animals, in this study, classified as adapted to heat and belonging to the same subspecies were grouped together. In all PC, genetic uniformity was observed for hybrid (taurine × indicine) animals adapted to heat conditions. For taurine animals, there was greater heterogeneity between and within populations. In all PC, greater heterogeneity was observed in the XIN population.

#### Genomic Inbreeding Coefficients

Inbreeding depression has been reported to affect reproductive and productive traits, longevity, health, and the ability of the individuals to cope with environmental challenges (Parland et al., 2009; Leroy, 2014; Pereira et al., 2016; Reverter et al., 2017; Doekes et al., 2019). Therefore, monitoring and controlling inbreeding is important to avoid inbreeding depression. The levels of inbreeding varied among populations and differed substantially among methods. Overall, the levels of genomic inbreeding were lower based on *F*_EH_ and *F*_ROH_, and higher based on *F*_HOM_. Inbreeding based on ROH is highly dependent on the ROH length, which can change according to the population analyzed (Rodríguez-Ramilo et al., 2019), sample size, and parameters used for the detection of ROH. MG, XIN, and YAK had the highest level of *F*_ROH_, a fact that can be attributed to the greater number of longer ROH regions.

Moderate correlation was observed between *F*_ROH_ and F_EH_, which can be explained by the fact that both *F*_ROH_ and F_EH_ directly reflect homozygosity levels in the genome (Zhang et al., 2015). Zhang et al. (2015) reported correlation between *F*_ROH_ and *F*_HOM_ (0.61) and between *F*_ROH_ and *F*_U_ (0.15), which are similar to the findings in this study. A similar correlation to our study (−0.07 versus −0.10) were found between *F*_HOM_ and *F*_U_ in Danish Red cattle and a negative correlation, but higher than found here (−0.07 versus −0.44), was observed between *F*_HOM_ and *F*_U_ in Danish HOL cattle bulls (Zhang et al., 2015). As discussed by Zhang et al. (2015), *F*_EH_ tends to be less accurate for populations with a high level of heterozygosity, as observed for SH, MG, KZK, and XIN. The authors indicated that a large sample size would be required to obtain a better estimate of genomic inbreeding in these populations. Solé et al. (2017) studying genomic inbreeding levels in Belgian Blue cattle found slightly higher (0.97) correlation between *F*_HOM_ and *F*_ROH_ than that found in this study (0.73). High correlation between *F*_HOM_ and *F*_ROH_ (0.85) has also been reported in other Chinese cattle breeds (Xu et al., 2019a). The differences in inbreeding coefficients can be related to sampling, sample size, accuracy of observed allele frequencies, statistical method used, and the SNP array (or genotyping platform) used.

The degree of relatedness between individuals within a population can be associated with ROH levels. Thus, population history events can also be inferred based on ROH analyses. In this context, short ROH are most likely correlated to ancestral inheritance or potential ancient bottleneck, whereas long ROH segments are more likely associated with relatively recent inbreeding (Purfield et al., 2012). The number of longer ROH in the MG breed may be attributed to more recent inbreeding. The same fact was observed for YAK, with the highest proportion of long ROH, but in this case it may also be due to the SNP chip used, as previously discussed.

#### LD and Consistency of Gametic Phase

The high average LD estimation for YAK are similar to those found in the literature. Wang et al. (2014) studying the genome variation between wild and domestic YAK using GBS found higher values of LD and lower LD decay for domesticated YAK than for wild YAK. Higher LD estimates have also been reported for Jinchuan yak (Wang et al., 2020). The higher LD values and lower LD decay pattern observed for YAK may be due to a potential bottleneck event during the domestication process (Wang et al., 2014).

The consistency of gametic phase evaluates the association between SNPs and QTL alleles across breeds (Brito et al., 2017). Thus, the linkage phase will not be consistent across populations over long distances in the genome if the genetic distance between populations is large. The consistency of gametic phase between breeds indicates whether or not different breeds could potentially be pooled into one common training population to better estimate SNP effects for genomic prediction of breeding values. The highest consistency of gametic phase was found between the hybrid cattle breeds, suggesting a greater level of relatedness between these populations. In dairy cattle, de Roos et al. (2009) reported that the benefits of combining populations in a training set were higher when the populations had diverged for only a few generations ago, when the marker density was high, and when the trait heritability was low.

In this study, the extent of LD at different distances between markers was computed and the ancestral effective population sizes was estimated. The use of LD pattern can be explored to comprehensively understand the population evolutionary history (Xu et al., 2019b). Knowledge of the effective size of a population can facilitate the design of selection schemes in animal breeding and the management of populations for endangered breeds or genetic lines (Mokry et al., 2014). Evaluating *N*e in Chinese cattle populations, Xu et al. (2019b) reported recent *N*e for South Chinese cattle populations (*N*e = 129) similar to the ones found in this study for DC, DH, Weiling Humped, and WN cattle. There was an increase in *N*e for YAK in the most recent generations (∼50 generations ago), which is probably due to the introduction of outbred animals or a high level of crossbreeding with other populations. To ensure a long-term viable genetic diversity, a threshold of *N*e equals to 100 has been suggested (Meuwissen, 2009). In our study, only three populations are above the threshold (Table 4), indicating that care should be taken in this regard to ensure that the *N*e and, consequently, reasonable genetic diversity levels are maintained.

#### PCA and Genomic Tree

The PCA showed a discrimination between heat adapted, cold adapted, YAK and BLI populations. As expected, the populations adapted to heat conditions clustered together as well as the cold ones, and YAK and BLI clustered separately (Figure 3). However, the first and third principal components showed an overlap among individuals from all different populations. These findings suggest that there is moderate genetic variability between the three groups and genetic similarity between populations clustered in the same group (i.e., heat and cold resilient and YAK). Especially on the first and third, and third and fourth components, it is possible to observe that XIN overlap with the BSW population which is also adapted to cold, and one of the breeds that XIN derived from Figures 5B,C. Another interesting cluster is between breeds from China (DH, DC, WN, WL) adapted to heat environments and are formed by crosses between taurines and indicine breeds. The *Bos taurus indicus* breeds formed another cluster, clearly visualized in both PCA scenarios. When considering just cattle population in the analysis (Supplementary Figure 1) the cluster from the previous scenario remains.

The genomic population tree constructed based on the genomic distance estimated between the cattle populations showed that there is differentiation between heat and cold adapted cattle populations. In this context, the breeds adapted to heat appear to be more related among themselves as well as those adapted to cold conditions. Our findings will be useful for policy makers, geneticists, and cattle breeders when making decisions toward the improvement of genetic resources, while conserving local breeds around the world.

### Signatures of Selection and Functional Analyses

Identifying signatures of selection contribute to better elucidate the mechanisms of selection to adaptation and climatic resilience and to identify candidate genes of interest to breeding programs and molecular studies. Using different methods, various genomic regions that are potentially under selection in at least one population or breed group were identified. Due to the fact that climate adaptation is a complex trait, influenced by several genes and metabolic pathways, the detection of only few loci under positive selection can be challenging (Kemper et al., 2014). The use of multiple methods reduces the chance of false positive results. In this study, there were many overlapping regions identified between at least two approaches (Supplementary Table 5), which provides stronger evidence of selection for specific traits (Hohenlohe et al., 2010; Oleksyk et al., 2010; Abied et al., 2020). It is worth mentioning that the use of different methods can result in the identification of different regions (Fariello et al., 2013), as methods might be based on different source of information (e.g., single-SNP allele frequency or haplotype structure). This grouping strategy facilitates the interpretation of the signatures of selection results and minimizes sources of variations due to selection for other group of traits.

Another important point is the use or not of outgroups when performing hapFLK analyses. The different genomic regions identified in both hapFLK scenarios might be due to the fact that an outgroup can provide the connection between groups of populations. As discussed by Brito et al. (2017), the use of single-SNP tests may fail to identify signals of selection when a single SNP is not in high LD with the causal mutation for the trait under selection. Another problem that may arise is that hapFLK and ROH may not capture ancient signatures of selection, for which the mutation-carrying haplotype is small and do not include many SNP on the SNP chip panel.

Despite the limitation of the ascertainment bias and reduced genomic coverage of the SNP dataset used, an interesting list of potential genes under selection in multiple cattle breeds, YAK, and BLI, which will be the foundation for future investigations could be still provided. For instance, the *RBPMS2* gene, found in YAK is related to age at first calving in Romosinuano and Costeño-con-Cuernos cattle breeds (Fernández et al., 2019) and the gene *ATG16L2* found in MG cattle is related to mastitis recovery in Danish HOL cows (Welderufael et al., 2017). Angrecka and Herbut (2016) observed that cows in excessive cold temperatures may show fertility impairments and the potential candidate gene *RBPMS2* found in YAK might be an indication of adaptation for the cold environments. Shathele (2009) observed a clear pattern of higher incidence of mastitis in dairy cows when the ambient temperature was lower than 21°C. The candidate gene *ATG16L2* found in the MG breed can be an indicator of cold adaptation. Also, the candidate gene *IL6ST* identified in KZK cattle, may be another indicator of fertility adaptation in cold environment, since this gene is implicated in uterine receptivity to conceptus implantation in sheep (Song et al., 2009). Two genes (*WARS2* and *TBX15*) were identified in ROH and were demonstrated by previous studies (Fumagalli et al., 2015; Hallmark et al., 2018) as having an important role in cold adaptation in Siberia Inuit (human) populations. Polymorphisms in or near *WARS2* and *TBX15* have been shown to be associated with numerous human phenotypes among individuals of European ancestry, including waist-hip ratio and fat distribution in Europeans (Heid et al., 2010). *TBX15* gene plays a role in the differentiation of brown (subcutaneous) and brite (typically inguinal) adipocytes, that in the case of exposure to cold can differentiate into cells capable of expressing uncoupling protein 1, producing heat by lipid oxidation (Gburcik et al., 2012). Therefore, *WARS2* and *TBX15* are candidate genes that might be associated with cold adaptation in both cattle and YAK.

The candidate gene *PLA2G2A* identified in the heat group in *F*_ST_1 is involved in lipid metabolism and was previously reported by Hallmark et al. (2018) in human inhabitants of Siberia, in which expression patterns was consistent with selection for cold climate and/or diet. The difference in adaptation environments found in this study and in theirs suggests that this candidate gene (*PLA2G2A*) may also be positively selected for lipid metabolism in warm environments. The gene *TMC6*, identified for mild cold/thermoneutral group based on *F*_ST_3 and ROH, has been reported to influence milk quality in cold environment in Northern China breeds (Yang et al., 2017). Thus, this gene may play an important role in adaptation to cold climate. Yang et al. (2017) also identified the gene *COL27A1* (found here in *F*_ST_2 and ROH), which has been proposed to be involved in cartilage calcification, which is another important aspect in cold climates. Studying signatures of selection for candidate genes for body temperature maintenance under the cold stress in Siberian cattle populations, Igoshin et al. (2019) found that the gene *GRIA4* contribute to cold-stress resistance due to indirect involvement in body thermoregulation. In our study a gene from the same family (*GRIA1*) was found for YAK in the *F*_ST_3 and ROH scenarios. This indicates that *GRIA1* may also be involved in cattle adaptation to cold climates. Howard et al. (2013) performed a GWAS for body temperature regulation in cattle during a five-day period of heat and cold stresses and identified candidate genes involved in vasculogenesis (*RASA1*) and ion regulation (*CACNG3, SLC22A23*, and *TRPC4*), which were also identified in our study for scenarios *F*_ST_2 and ROH (*RASA1*), and *F*_ST_3 and ROH (*CACNG3*), *F*_ST_3 and ROH (*SLC22A23*), and *F*_ST_1 and ROH (*TRPC4*).

High altitudes, where YAKs are raised, might result in hematopoiesia, where the reduced oxygen tension leads to an increase of erythrocytes as an adaptive mechanism to low oxygen levels (Jain, 1993; Ding et al., 2014). The candidate genes *HBM* and *HBG*, related with oxygen transportation were identified for YAK in *F*_ST_1, *F*_ST_2, and ROH (*HBM*) and in hapFLK and ROH (*HBG*). These genes were previous identified by Mehla et al. (2014) in a GWAS of heat stress response in Zebu (Sahiwal) cattle. In their study, *HBM* and *HBG* were suppressed in the presence of heat stress. The fact that these genes were found here as under positive selection in YAK highlights the importance of these genes in oxygen transportation in harsh and high-altitude environments.

The genes *SLC38A4*, *AGXT2*, *STEAP3*, *CSMD3*, *ABCB4*, *ZDBF2*, and *LAMA2* identified in *F*_ST_1 and ROH, were previously reported in a study of functional characterization of mammalian genes involved in adaptation to Arctic or Antarctic environments (Yudin et al., 2017) and had traces of positive selection in human, mammoth, and polar bear (*SLC38A4*), mammoth and polar bear (*AGXT2*, *STEAP3*, *CSMD3* and *ABCB4*), mammoth, whale, and polar bear (*ZDBF2*), and human and mammoth (*LAMA2*). The gene *SLC38A4* encodes a transporter protein that is expressed predominantly in the liver and participates in the transfer of amino acids (Bröer, 2013) and is potentially involved in gluconeogenesis (González-Renteria et al., 2013). Regarding the genes *AGXT2* and *STEAP3*, Yudin et al. (2017) reported that these genes had two enriched terms related to regulation of reactive oxygen species metabolic process and transition metal ion transport. Similar to the *AGXT2* and *STEAP3* genes, Yudin et al. (2017) reported *ABCB4* to be associated with the enriched term bile acid secretion, and likely related to fat-rich diets of mammals in the Arctic and Antarctic regions. The gene *ZDBF2* was paternally expressed in lymphocytes, but bi-allelically expressed in the placenta in humans (Kobayashi et al., 2009) and related to various genetic abnormalities (Petit et al., 2004). The positive selection in the *ZDBF2* gene might be related with physiological or immunological adaptations of animals to low temperatures. These findings represent a strong indication of the role of these genes (*SLC38A4*, *AGXT2*, *STEAP3*, *ABCB4*, *ZDBF2*, and *LAMA2*) in the metabolism and resistance of animals exposed to harsh environments (e.g., low temperatures and high altitudes).

The stress caused by high temperatures has a great impact on the animal’s life and can induce behavioral and metabolic changes in cattle that are intended to maintain homeothermy, often at the expense of decreased productivity, welfare, health, and profitability (West, 1994; Gao S. T. et al., 2017). In Weiling Humped cattle, which is adapted to hot environments, the gene *HSPE1*, which regulates maintenance of metabolic activity and survival (Kumar et al., 2015) was identified. Pitt et al. (2018), studying the local adaptation of Creole cattle genome diversity in the tropics, reported an association between *SNORA70* with feed intake, body conformation, and live weight. Here this gene was identified in populations adapted either to cold or heat conditions, indicating its importance for metabolic adaptation in both thermal conditions. In DH and DC cattle, both adapted to hot environments, the gene *EIF4E* was identified, which is associated with conceptus development and/or return to cyclicity in cattle (Forde et al., 2011). The gene *SNORD116* identified in DC cattle, has been reported to regulate food intake and body weight in mice (Qi et al., 2016). Three genes (*LOX*, *HSPB9*, and *CSF3*) identified based on *F*_ST_1, *F*_ST_3, and ROH in DH were previous reported to harbor selective sweeps for environmental adaptation in EAZ (Bahbahani et al., 2017). Another identified gene was the *DNAJC8* which is a member of the DnaJ family which is known to act as cofactors for other heat shock proteins (i.e., HSP70) to maintain protein folding under heat stress (Coleman et al., 1995; Kampinga and Craig, 2010). The *LOX* gene, on the other hand, is important to maintain optimum growth and development and it is associated with lung and blood vessels (Mäki et al., 2005). The *CSF3* gene was related with innate and adaptive immune response in African cattle, as this gene acts as a positive regulator for macrophages to induce their antimicrobial effects (Bronsvoort et al., 2013; Bahbahani et al., 2017). The presence of this gene in African cattle was expected since they are exposed to a diversity of pathogens and physiological stressors in their surrounding environment, e.g., endoparasites, haemoparasites, and bacteria (Bronsvoort et al., 2013; Murray et al., 2013; Thumbi et al., 2014).

Two genes identified between *F*_ST_1 and ROH (*NRXN3* and *CACNB2*) were previously reported to be related to fertility traits in cattle (Reverter et al., 2017). *NRXN3* harbors a large number of mutations associated with heifer puberty in cattle (Dias et al., 2017). *CACNB2* is related to the secretion of the follicle stimulating hormone, and variants of this gene have been found to be associated with conception rate in cows (Sugimoto et al., 2013). Reverter et al. (2017) also identified *XKR4* as an important candidate gene for climate adaptation in Australian beef cattle. This gene (*XKR4*) was previously reported to be associated with fat deposition (Porto-Neto et al., 2014; Silva et al., 2017), feed intake and growth (Lindholm-Perry et al., 2011) phenotypes. The genes *SLC9A4*, *PLCB1*, *FTO*, *ITPR2* identified in several scenarios for the heat adaptation group were previously reported by Taye et al. (2017) as under positive selection for thermotolerance in African cattle. *SLC9A4* and *ITPR2* are involved in cellular process in thermal sweating in African cattle. *ITPR2* promotes the release of calcium from extracellular interstitial fluid and release of intracellular Ca_2_^+^ stores needed for normal sweat production (Cui and Schlessinger, 2015) and *SLC9A4* is Na^+^/H^+^ exchangers that are expressed in the duct and secretary sweat glands in humans (Cui and Schlessinger, 2015). Another gene identified by Taye et al. (2017) is the *PLCB1* gene, which hydrolyzes phospholipids into fatty acids and other lipophilic molecules. *PLCB1* was previously reported as positively selected in sheep and goats in response to adaptation to dry-arid environments (Kim et al., 2015). Taye et al. (2017) also found the *FTO* gene as related to metabolism and feed intake, being *FTO* a nuclear protein of the AlkB related nonhaem iron and 2-oxoglutarate-dependent oxygenase superfamily, which is known to contribute to the regulation of the global metabolic rate, energy expenditure, energy homeostasis and feed intake (Zhang et al., 2011; Zielke et al., 2013). The above-mentioned genes (*SLC9A4*, *PLCB1*, *FTO*, *ITPR2*) might contribute to improved production and reproduction under heat stress conditions.

Immune and anti−infectious response related genes were identified in *F*_ST_1 and ROH (*LEF1* and *MLST8*), and *F*_ST_2, *F*_ST_3, and ROH (*SMYD3*). Flori et al. (2019) indicated that *LEF1*, *MLST8*, and *SMYD3* are genes related with immune and anti−infectious response in Mediterranean cattle breeds. *LEF1* could be a strong candidate gene to heat adaptation since it is also related to thermotolerance and ultra-violet protection, as suggested by its central role in hair pigmentation (Gautier et al., 2009). Studying environmental adaptation of indigenous Chinese cattle, Gao Y. et al. (2017) also identified the gene *LEF1* near several known color genes. *TICAM1* and *CXCR4* genes (identified in *F*_ST_1 and ROH) are involved in antigen recognition and related to the development of immune response in West African cattle (Gautier et al., 2009). *TICAM1* possesses a high interferon type I inducing activity during viral infection and thus plays a role in innate immunity (Seya et al., 2005; Gautier et al., 2009) and *CXCR4* is related to antigen presentation to T-cells in the context of major histocompatibility complex, a dense region of genes in the mammalian genome involved in the immune system, autoimmunity, and reproductive success (Contento et al., 2008; Gautier et al., 2009). The presence of these genes related to the immune response in populations adapted to heat highlights the importance of these genes to the immune response of animals during heat stress events.

**FIGURE 9:**
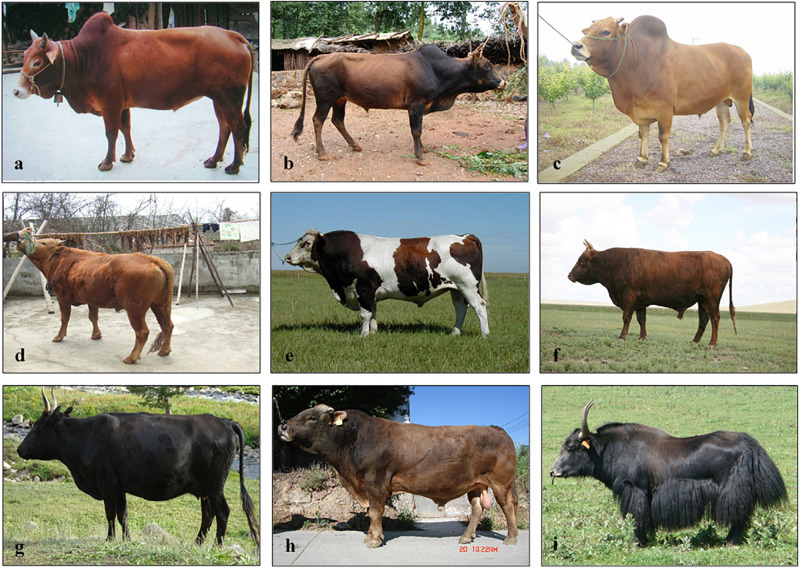
Local Chinese cattle populations and yak: **(a)** Yunnan Humped; **(b)** Dengchuan; **(c)** Wenling Humped; **(d)** Wannan; **(e)** Sanhe; **(f)** Mongolian; **(g)** Hazake; **(h)** Xinjiang Brown; and **(i)** Datong Yak. Photo credits: Animal genetic resources in China: Bovines.

A slick coat is associated to higher thermotolerance and higher milk production on crossbred animals under tropical environment and can be considered as an indicator or indirect phenotype to some highly important production traits (Dikmen et al., 2014; Casas and Kehrli, 2016; Carabaño et al., 2017; Porto-Neto et al., 2018). A deletion within the last exon of *PRLR* gene that truncates a portion of the cytoplasmic domain of the protein is responsible for this very smooth coat and were first identified in the SEN breed (Flori et al., 2012; Littlejohn et al., 2014). The *PRLR* gene was also identified in our study (based on *F*_ST_1 and ROH) in WN and SEN, breeds with short coat hair. This finding in WN might be linked to thermotolerance in this cattle breed, an adaptation to high temperature environment. Another important trait in thermal tolerance adaptation in livestock is coat color (Mcmanus et al., 2008; Abdurehman, 2019). Cows with light coat colors in tropical regions reflect solar radiation; thereby protecting the animal from the adverse effects of solar radiation (Fanta, 2017; Gaughan et al., 2018). The gene *PMEL*, identified in *F*_ST_1 and ROH in DC cattle (which is characterized by a yellow coat; Table 1 and Figure 9), is essential for melanosome development and is responsible for lightening or diluting the base color defined by the Extension locus (*MC1R*) in some cattle breeds (Schmutz and Dreger, 2012; Flori et al., 2014). The positive selection identified in DC cattle might be due to the association of this gene (*PMEL*) with thermotolerance.

Heat shock factors have the function to act as chaperones assisting in protein folding, thereby avoiding protein aggregation and, as result, protein homeostasis during cellular response to heat stress (Moseley, 1997; Hightower, 2004). In our study, gene members of the heat shock family were identified (*DNAJA1*, *DNAJB1*, *HSPH1*, and *HSPA4*). Srikanth et al. (2016), studying genes and pathways linked to heat stress in HOL calves, identified 20 upregulated genes encoding molecular chaperones in response to heat stress. Among them, the members of HSP40 (*DNAJA1* and *DNAJB1*) that regulates the ATPase activity of HSP70 (*HSPH1* and *HSPA4*) by interacting with the J domain of the HSP70 proteins and also conferring thermo-tolerance to cells on exposure to heat stress (Maio, 1999). O’Brien et al. (2014), assessing signatures of selection through variation in linkage disequilibrium between taurine and indicine cattle, found the gene *DNAJA1* in BSW and ANG cattle. A previous study (Bernard et al., 2007) has reported *DNAJA1* to be linked to meat tenderness, since this gene explained up to 63% of the phenotypic variability of tenderness in Charolais cattle. In our study, *DNAJA1* was also found for SH cattle, which are animals with good meat quality, and thus, *DNAJA1* might be a good indicator of selection for meat tenderness in this breed.

The high number of genes identified in our study (Tables 5, 6 and Supplementary Material) makes it challenging to prioritize the most important candidate genes. The majority of genes were linked to resilience, adaptation response, health, fertility, and production. Important biological pathways and functions have been identified and have important functions related to adaptation to thermal stress and can help to identify possible candidate genes. Four genes (*ALB*, *HBM*, *HBZ*, and *HBQ1*) were clustered in the “oxygen binding” molecular function that was identified for YAK in scenarios SCEN1 and SCEN4. At high altitudes, where YAKs are raised, immediate means of acclimatization such as hyperventilation, hemoconcentration, and erythropoiesis are initiated to increase total oxygen carrying capacity (Burtscher et al., 2018). In such environments, it is observed increased oxygen binding by hemoglobin even at low arterial partial oxygen pressure (Mairbäurl and Weber, 2012; Burtscher et al., 2018). In addition, the candidate genes *HBM*, *HBZ*, and *HBQ1* were grouped in other two clusters identified for YAK, which are related to oxygen transport and hemoglobin complex, indicating that these three genes may be of interest when selecting animals adapted to high altitudes where the oxygen level is lower and biological adaptations are required. Three candidate genes (*FOLR1*, *FOLR2*, and *FOLR3*) were grouped into two groups related to folate receptor and folic acid transporter activity. Koyama et al. (2012), studying the effects of folic acid on the development and oxidative stress of mouse embryos exposed to heat stress found that the inhibitory effects of heat stress on the development of mouse pre-implantation embryos are ameliorated by folic acid.

Heat stress can be considered as cytotoxic, as it alters biological molecules, disturbs cell functions, modulates metabolic reactions, induces oxidative cell damage, and activates both apoptosis and necrosis pathways (Pandey et al., 2012; Slimen et al., 2015). Four genes (*NDUFS3*, *NDUFB6*, *CYCS*, and *CYCT*) were clustered together and are related to respiratory chain. Ganaie et al. (2013) and Nisar et al. (2013) suggested that heat stress is responsible for inducing oxidative stress during summer in livestock animals, which enhances reactive oxygen species production and induces oxidative stress, what can lead to cytotoxicity (Slimen et al., 2015). The identification of these four genes related to respiratory chain in the scenarios of animals adapted to heat stress might indicate that these genes play an important role in preventing oxidative stress and cytotoxicity caused by high temperatures. High temperature is also considered to be one of the most important factors for subfertility in cattle (Takahashi, 2011; Das et al., 2016). Three genes (*BSP1*, *BSP3*, and *BSP5*) related to fertilization and sperm capacitation were identified in scenario SCEN2, based on the heat adaptation candidate genes group. Manjunath and Thérien (2002) reported the binder of sperm protein (BSP) family as a key step to sperm capacitation in ejaculation, as BSP bind to sperm and induce efflux of membrane cholesterol and phospholipids (Therrien et al., 2013). The BSP family genes identified here might indicate that under heat stress these genes play an important role on sperm capacitation and consequently, bull fertility.

The large number of genomic regions and candidate genes identified in this study was not unexpected. As there are multiple mechanisms involved in cold and heat stress response, natural and artificial selection are expected to cause weak selection pressure at many sites across the genome. The weak selection pressure is due to the small effect of each of the many variants or QTL underlying the phenotype, which results in small changes to allele frequencies at the many loci, but which result in the adaptation response observed across breeds (Kemper et al., 2014).

The information reported here will be useful for the implementation of genomic selection and other genomic studies in local Chinese cattle populations and YAK. Genomic selection is expected to increase the genetic gain along with the decrease in generation interval (Farah et al., 2015; Kariuki et al., 2017; Mehrban et al., 2017; Weller et al., 2017; Doublet et al., 2019). Due to the decrease in generation interval, a decrease in genetic diversity could be expected (Doublet et al., 2019). Eynard et al. (2018) indicated that optimal contribution strategy is an alternative to avoid the loss of genetic diversity and obtain genetic gain, as it takes into account both the relationships and genetic merit of the individuals. Further investigation using additional breeds, more complete datasets (e.g., larger number of breeds and phenotypes), and larger numbers of SNP are recommended to validate the role and the specific function of the highlighted candidate genes associated with climate adaptation in this study. Alternative “-omic” technologies (e.g., transcriptomics) and functional studies should be conducted to validate the function of the candidate genes identified.

Lastly, some of the potential limitations of this study should be highlighted. Having additional breeds from the same subspecies, but adapted to different climatic conditions, would be beneficial. Ascertainment bias might have influenced the estimates due the fact the SNP array design did not include all the cattle populations evaluated and especially any population of YAK or BLI. Moreover, due to the large number of candidate genes identified, additional studies should prioritize specific genes for more in-depth studies for validation purposes.

## Conclusion

A comprehensive description of genetic diversity measures in thirty-two worldwide cattle populations (with a focus on Chinese local breeds), Datong yak, and Bali populations is presented. Moderate genetic diversity was observed within each population, but optimal contribution strategies should be adopted to avoid further reduction in the genetic diversity of these populations. The breeds adapted to heat seems to be moderately related to each other and the same was observed for those adapted to cold environments. This study provides a high-confidence map of positive selection signatures for thermal tolerance in cattle, and Datong yak using three alternative methods (*F*_ST_, hapFLK, and ROH). Various candidate genes were identified as potentially under selection for thermal tolerance based on multiple methods and scenarios. Enrichment analyses identified important biological pathways, molecular function and cellular components, which greatly contribute to our understanding of the genetic background of thermal tolerance in cattle and Datong yak. These results highlight the diversity and complexity of mechanisms that are involved in response to harsh environmental conditions. Further studies are needed to validate these results by using larger and independent populations, additional methods for specific thermal stress indicators (e.g., GWAS), and other tools such as whole-genome sequencing. These novel findings are useful for the conservation of genetic resources, especially under global climate changes, and contribute to the potential discovery of functional genes and genetic mechanisms related to thermal tolerance in cattle, Datong yak, and Bali populations.

## Data Availability Statement

All relevant information supporting the results of this article are included within the article and its additional files. Public genotypic data used for our analysis can be found at WIDDE database: http://widde.toulouse.inra.fr/widde/widde/main.do;jsessionid=73509E5BFFDDE224635B91CA70E9FC82?module=cattle.

## Author Contributions

LB, YW, QX, and FS participated in the design of the study. PF performed all the analyses and results interpretation, was involved in the discussions, and wrote the first draft of the manuscript. LB provided training to the first author, contributed to the analyses and results interpretation, was involved in all the discussions, and edited all the versions of the manuscript. YW and YZ provided the cattle genotypes, while PY provided the YAK genotypes used for the study. HO participated in the results interpretation, was involved in the discussions and gave editorial assistance. FS, YW, QX, and PY edited the final version of the manuscript and provided valuable suggestions. All authors have approved the final manuscript.

## Conflict of Interest

The authors declare that the research was conducted in the absence of any commercial or financial relationships that could be construed as a potential conflict of interest.

## Footnotes

1 http://useast.ensembl.org/index.html

2 www.ncbi.nlm.nih.gov/gene
