# Supplementary material for: Genetic Diversity and Signatures of Selection for Thermal Stress in Cattle and Other Two Bos Species Adapted to Divergent Climatic Conditions
Source: Front Genet. 2021 Feb 3;12:604823. doi: 10.3389/fgene.2021.604823 (PMC7887320; doi:10.3389/fgene.2021.604823)
Supplement: Supplementary file 2 [file Presentation_1.pdf]

SUPPLEMENTARY FIGURES

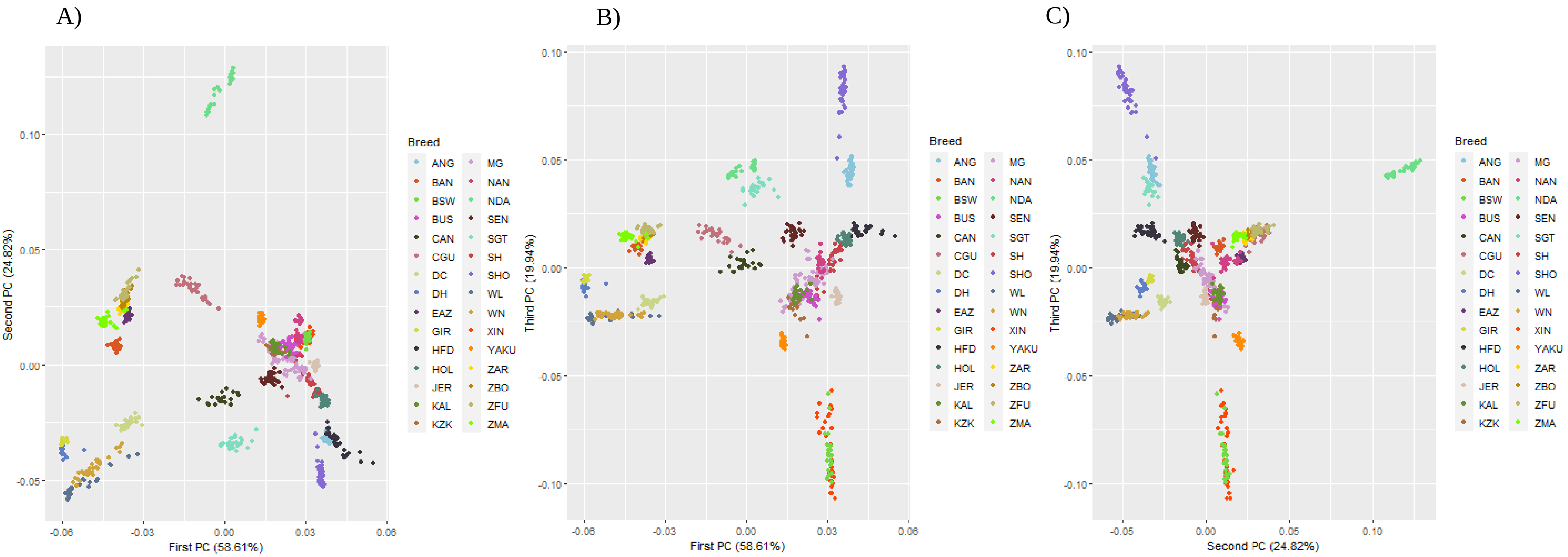

Figure S1. Principal component decomposition of the genomic relationship matrix colored by population*. Letters in the figure represent the decomposition of the: **A)** first and second; **B)** first and third; and **C)** second and third principal components, respectively. (*see Table S1 in the Supplementary Materials for abbreviations)

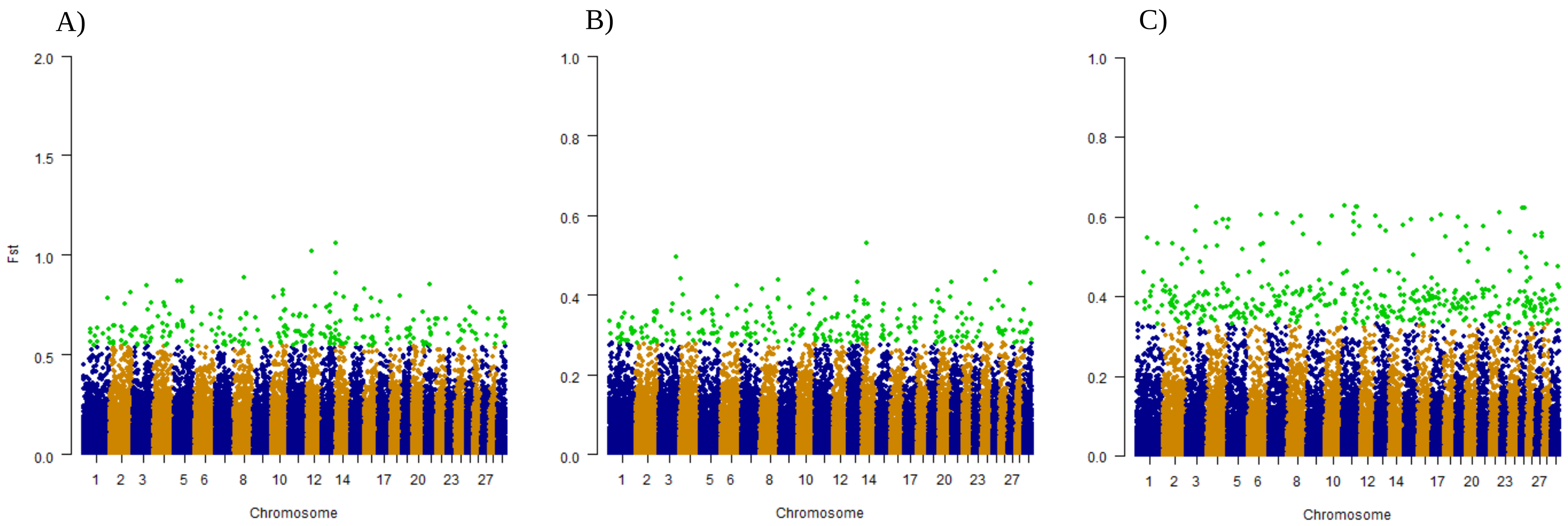

Figure S2. SCENARIO 2: Whole genome scans for signatures of selection using the FST approach for 32 cattle breeds and yak, considering climate adaptation. Letters in the figure represent: **A)** Heat adapted populations (Yunnan Humped, Dengchuan, Wenling Humped, Wannan, Arabic zebu, Bangladesh zebu, Boran, Canchin, Creole from Guadalupe, East African Shorthorn Zebu, Gir, N'dama, Senepol, Zebu Bororo, Zebu from Madagascar and Zebu Fulani); **B)** Cold/thermoneutral adaptation populations (Sanhe, Mongolian, Hazake, Xinjiang Brown, Angus, Hereford, Holstein, Jersey, Brown Swiss, Kalmykian, Negra Andaluza, Santa Gertrudis and Shorthorn) and **C)** Harsh cold populations (Busha, Yanbian and Yakutian).

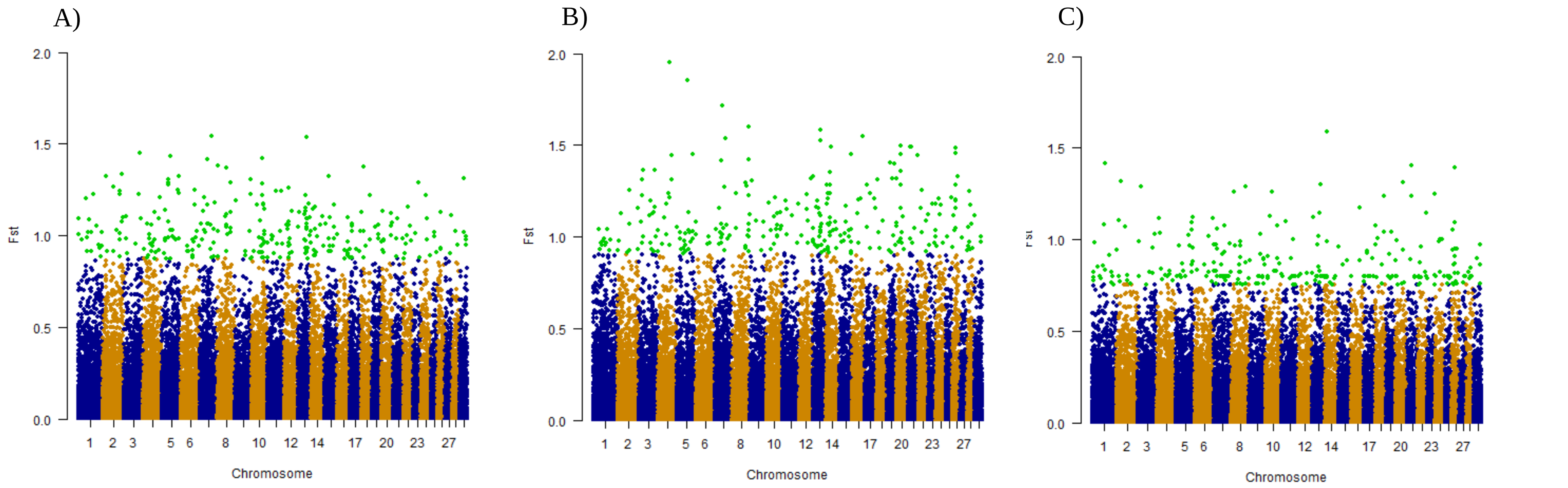

Figure S3. SCENARIO 3: Whole genome scans for signatures of selection using the FST approach for 32 cattle breeds and yak, considering climate adaptation. Letters in the figure represent: **A)** Heat adapted populations (Yunnan Humped); **B)** Cold/thermoneutral adaptation populations (Holstein) and **C)** Harsh cold populations (Yakutian).

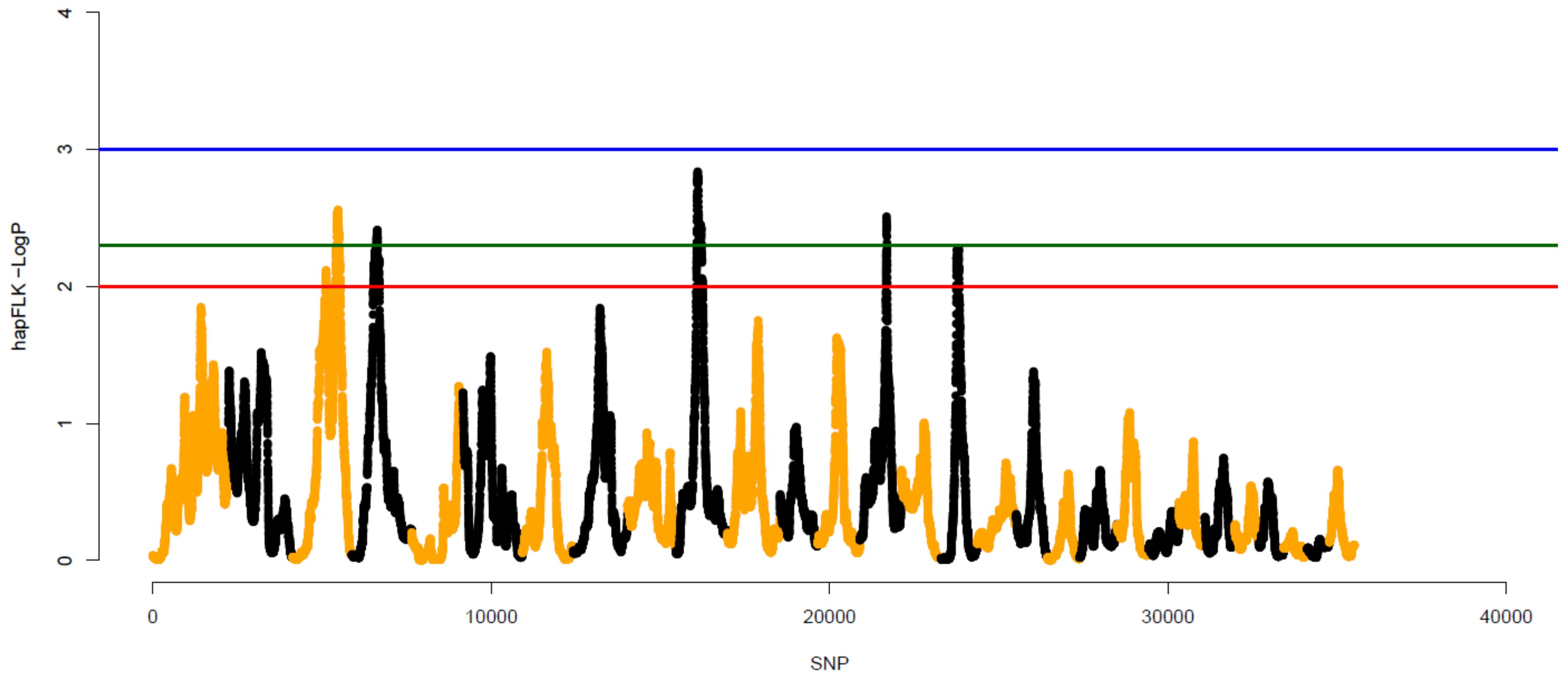

Figure S4. Whole genome scans for signatures of selection using the haplotype based hapFLK metric considering 32 cattle breeds and Bali as outgroup (second scenario). Odd and even numbered chromosomes are shown in yellow and black, respectively. SNP number is given on the x axis, and the genome-wide threshold corresponding to $P < 0.001$, $P < 0.005$ and $P < 0.01$ is shown as horizontal blue, green and red lines, respectively.

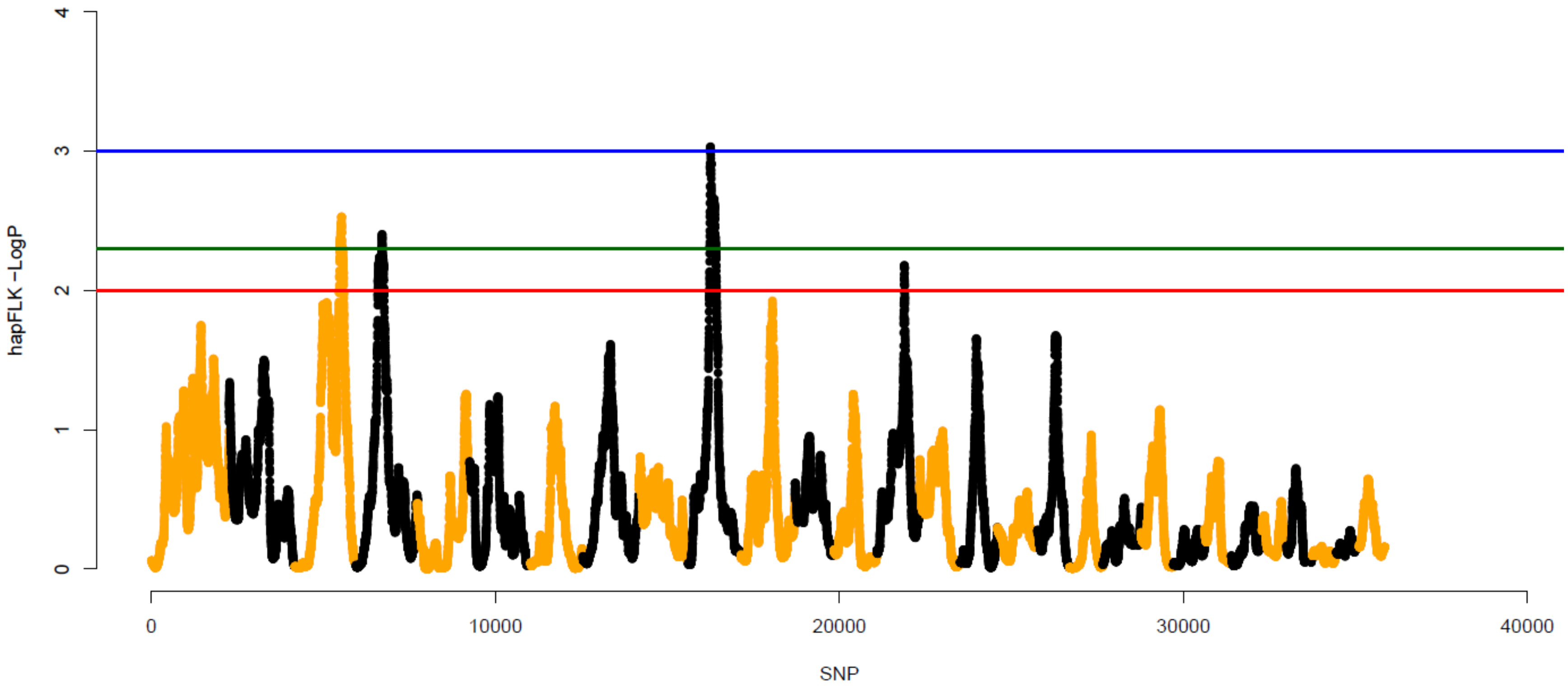

Figure S5. Whole genome scans for signatures of selection using the haplotype based hapFLK metric considering 32 cattle breeds and no outgroup (third scenario). Odd and even numbered chromosomes are shown in yellow and black, respectively. SNP number is given on the x axis, and the genome-wide threshold corresponding to $P < 0.001$, $P < 0.005$ and $P < 0.01$ is shown as horizontal blue, green and red lines, respectively.
